# Effects of exercise on reproductive endocrine hormones in adult patients with polycystic ovary syndrome: a systematic review and three-level meta-analysis

**DOI:** 10.7717/peerj.21507

**Published:** 2026-07-29

**Authors:** Xiao Chen, Yongjie Liu, Duohui Chen, Lijun Chen

**Affiliations:** 1 Shanghai Zhongqiao Vocational and Technical University, Shanghai, China; 2 Jiaxing University, Jiaxing, Zhejiang Province, China; 3 Shanghai Polytechnic University, Shanghai, China; 4 Shanghai Lixin University of Accounting and Finance, Shanghai, China

**Keywords:** Exercise, Polycystic ovary syndrome (PCOS), Endocrine hormones

## Abstract

**Objective:**

This study aims to systematically evaluate the impact of exercise interventions on reproductive endocrine profiles in adult patients with polycystic ovary syndrome (PCOS) using a three-level meta-analytical approach.

**Methods:**

We performed a systematic search of PubMed, Web of Science, The Cochrane Library, and Embase for randomized controlled trials (RCTs) investigating the effects of exercise on reproductive endocrine profiles in PCOS patients, spanning from database inception through April 7, 2026. A three-level meta-analysis was conducted in R using a random-effects model fitted *via* restricted maximum likelihood (REML). Effect sizes were quantified as Hedges’ g with 95% confidence intervals (CIs). Global heterogeneity was assessed using the Cochran’s Q test, and the robustness of results was verified through leave-one-out sensitivity analyses. Model fit was compared using likelihood ratio tests (LRT). Publication bias was scrutinized *via* Egger’s test, with the trim-and-fill method employed as a corrective measure where necessary. Methodological quality was appraised using the PEDro scale, and the certainty of evidence was graded *via* the GRADE profiler.

**Results:**

Nineteen studies involving 1,018 participants were synthesized. Exercise interventions significantly lowered testosterone levels (g = −0.34; 95% CI [−0.61 to −0.07]; *P* = 0.0146). Subgroup analysis revealed that the “aerobic exercise” group was statistically significant (k = 14, g = −0.5120, 95% CI [−0.8230 to −0.2010], *P* = 0.0027). The mean body mass index (BMI) “25 to 29.9” group was also statistically significant (k = 17, g = −0.4859, 95% CI [−0.7775 to −0.1943], *P* = 0.0025), as was the mean age group of “26 to 30 years old” (k = 13, g = −0.4878, 95% CI [−0.8465 to −0.1290], *P* = 0.0105).

**Conclusions:**

In conclusion, exercise interventions exert a favorable impact on serum testosterone (T) levels in adult patients with polycystic ovary syndrome (PCOS).

**Other:**

PROSPERO (registration no. CRD420251108464; https://www.crd.york.ac.uk/prospero/).

## Introduction

Polycystic ovary syndrome (PCOS), one of the most prevalent endocrine disorders affecting adult women, is clinically defined by chronic anovulation, hyperandrogenemia, and polycystic ovarian morphology (Gnanadass, Prabhu & Gopalakrishnan, 2021). According to the International Evidence-based Guidelines for the Assessment and Management of PCOS (2023), the prevalence among women of reproductive age ranges from 10% to 13% (Teede et al., 2023). Projections indicate that by 2036, the age-standardized incidence rate (ASIR) and prevalence rate (ASPR) of PCOS will increase by 8.32% and 10.87%, respectively (Wang et al., 2025). Clinically, PCOS manifests with menstrual irregularities, infertility, and elevated androgen levels (Xu et al., 2023), profoundly impairing female reproductive function (Xu & Qiao, 2022), and is associated with increased risks of ovarian and endometrial cancers (Stener-Victorin et al., 2024).

Pharmacological treatment remains the primary approach but carries side effects, such as potential memory impairment from long-term metformin use and visual disturbances induced by clomiphene (Alhowail, 2021; Kim et al., 2023). Lifestyle modification, encompassing dietary changes and exercise interventions, is recognized as a first-line non-pharmacological treatment for PCOS (Gu et al., 2022; McGowan et al., 2025; Milton, Gomersall & Schipperijn, 2023; Zhang et al., 2023). Nevertheless, dietary modifications are often more effective when combined with pharmacotherapy and show limited long-term benefits when used alone (Barrea et al., 2019; Tay et al., 2020). Evidence supporting physical exercise as an effective and increasingly accepted therapy for PCOS is growing (Butt et al., 2023; Mohamed et al., 2025). Exercise substantially reduces insulin resistance (Jiang, Chen & Huang, 2025), improves sexual function, reduces anxiety and depression-related indices, enhances body image perception (Kogure et al., 2020), and positively modulates reproductive endocrine hormone levels (Qiu et al., 2023; Slojewska et al., 2024).

A meta-analysis investigating the effects of exercise on reproductive endocrine hormones in patients with PCOS found no statistically significant effect of exercise on testosterone (T) levels in this population (Kelly dos Santos et al., 2020). While the included studies reported no statistical differences at baseline between the intervention and control groups, estimating effect sizes solely from post-intervention values may introduce potential biases. The absence of statistical significance in baseline differences does not imply complete equivalence between the two groups; especially in studies with small sample sizes or limited statistical power, minor but clinically significant baseline imbalances may remain undetected. These unrecognized baseline differences could still impact intergroup comparisons at the intervention’s conclusion, blending existing differences with intervention effects and thus limiting the accuracy of causal interpretations. To address these gaps, we expanded upon existing syntheses by incorporating 14 additional trials, resulting in a total of 19 studies encompassing 1,018 participants (intervention: *n* = 595; control: *n* = 423). We utilized a three-level hierarchical meta-analysis to account for nested effect sizes within individual studies, thereby maximizing data preservation and statistical robustness. Furthermore, we examined how various moderators—including exercise modality, intensity, duration, baseline body mass index (BMI), age, and blood sampling timing—influence reproductive endocrine profiles (primary outcome) and BMI (secondary outcome). These findings are intended to inform the development of precision exercise prescriptions and evidence-based clinical management for adult PCOS patients. The reproductive endocrine hormones examined in this study include testosterone (T), follicle-stimulating hormone (FSH), luteinizing hormone (LH), estradiol (E_2_), anti-Müllerian hormone (AMH), progesterone (P), and prolactin (PRL).

## Subjects and methods

### Inclusion and exclusion criteria

Inclusion criteria were as follows (Hutton et al., 2015): (1) Study Participants: Patients diagnosed with PCOS according to the Rotterdam Consensus or by hospital standards (Fauser et al., 2004), aged over 18 years, with no restrictions on race or country of origin; (2) Intervention Measures: Any form of physical exercise aimed at improving health, characterized by specific intensity, frequency, and duration of structured physical activity (Xie et al., 2024). This includes both single exercises and combinations of multiple exercise types. There are no restrictions on the duration of the intervention, and supervision is optional; (3) Control Conditions: The control group acts as a blank control, maintaining their usual lifestyle without engaging in any physical exercise or receiving any non-exercise-related interventions.; (4) Outcome Indicators: The study must include at least one measure of body mass index (BMI) and one reproductive endocrine hormone. The reproductive endocrine hormones to be considered include testosterone (T), follicle-stimulating hormone (FSH), luteinizing hormone (LH), estradiol (E_2_), anti-Müllerian hormone (AMH), progesterone (P), and prolactin (PRL); (5) Study Type: Randomized controlled trials (RCTs) involving adult female participants.

Exclusion Criteria: 1. Reviews, conference proceedings, editorial comments, animal studies, and duplicate publications; 2. Non-RCTs and study protocols; 3. Studies with inaccessible full texts, incomplete data, data that cannot be converted, or failure to obtain original data after contacting authors; 4. Outcomes not included in this study; 5. Interventions that are not solely physical exercise, potentially involving lifestyle modifications and/or pharmacological treatments.

### Literature search

The literature search covered the period from the inception of each database through April 7, 2026. A comprehensive search strategy was employed, integrating titles, abstracts, and subject headings, and tailored to the specific indexing features of each database. The databases queried comprised PubMed, Web of Science, The Cochrane Library, and Embase. PubMed serves as an essential component of the core literature resources offered by the National Library of Medicine (NLM) in the United States, covering a diverse array of fields such as life sciences, medicine, and nursing. Web of Science (WoS) is internationally recognized as a reputable academic search platform, with searches conducted within its core collection, which comprises sub-databases such as the Science Citation Index (SCI) and the Social Science Citation Index (SSCI). The Cochrane Library serves as a pivotal resource for international collaboration in evidence-based medicine, focusing on the identification of high-quality systematic reviews and clinical trials. Embase is well-known for its extensive coverage of literature in domains such as drug adverse reactions and pharmacological research. The search strategies utilized by these databases conform rigorously to academic standards, ensuring both comprehensive and representative literature coverage. Detailed search strategies are provided in Table 1.

**Table 1:** Search strategy.

Data base	Retrieval procedure
PubMed	#1 “Polycystic Ovary Syndrome”[Mesh]OR“Polycystic Ovarian Syndrome” [Title/Abstract]OR“Sclerocystic Ovarian Degeneration”[Title/Abstract]OR“Stein Leventhal Syndrome”[Title/Abstract]OR“Sclerocystic Ovaries”[Title/Abstract]OR“Ovary, Sclerocystic”[Title/Abstract]OR“Sclerocystic Ovary”[Title/Abstract]OR“PCOS”[Title/Abstract] #2“Exercise”[Mesh]OR“Exercise,Physical”[Title/Abstract]OR“Physical Exercise”[Title/Abstract]OR“Exercise,Aerobic”[Title/Abstract]OR“Aerobic Exercise”[Title/Abstract]OR“Exercise, Isometric”[Title/Abstract]OR“Isometric Exercise”[Title/Abstract]OR“Acute Exercise”[Title/Abstract]OR“Exercise, Acute”[Title/Abstract]OR“Exercise Training”[Title/Abstract]OR“Training,Exercise”[Title/Abstract]OR“Physical Activity”[Title/Abstract]OR“Activities, Physical”[Title/Abstract]OR“Activity,Physical”[Title/Abstract]OR“Physical Activities”[Title/Abstract]OR“Running”[Title/Abstract]OR“Jogging”[Title/Abstract]OR“Fitness”[Title/Abstract]OR“Yoga”[Title/Abstract]OR“Exergaming”[Title/Abstract] OR“Fit aerobics”[Title/Abstract]OR“HIIT”[Title/Abstract]OR“High-Intensity Interval Training”[Title/Abstract]OR“Swim”[Title/Abstract]OR“Walking”[Title/Abstract]OR“Hiking”[Title/Abstract]OR“Stair Climbing”[Title/Abstract]OR“Racquet Sports”[Title/Abstract]OR“Badminton”[Title/Abstract]OR“Tennis”[Title/Abstract]OR“Dancing”[Title/Abstract]OR“Qigong”[Title/Abstract]OR“Tai Ji”[Title/Abstract]OR“Contrology”[Title/Abstract]OR“cycling”[Title/Abstract]OR“Strength”[Title/Abstract]OR“Resistance”[Title/Abstract] #3 “Body Mass Index”[Mesh]OR“Quetelet Index”[Title/Abstract]OR“BMI”[Title/Abstract]OR“Follicle Stimulating Hormone”[Mesh]OR“Follitropin”[Title/Abstract]OR“FSH”[Title/Abstract]OR“Luteinizing Hormone”[Mesh]OR“Hormone, Luteinizing”[Title/Abstract]OR“ICSH”[Title/Abstract]OR“Interstitial Cell Stimulating Hormone”[Title/Abstract]OR“Hormone, Interstitial Cell-Stimulating”[Title/Abstract]OR“Lutropin”[Title/Abstract]OR“Luteozyman”[Title/Abstract]OR“Luteoziman”[Title/Abstract]OR“LH”[Title/Abstract]OR“Estradiol”[Mesh]OR“Oestradiol”[Title/Abstract]OR“Estraderm TTS”[Title/Abstract]OR“Estrace”[Title/Abstract]OR“Ovocyclin”[Title/Abstract]OR“Progynova”[Title/Abstract]OR“Progynon Depot”[Title/Abstract]OR“Delestrogen”[Title/Abstract]OR“Aerodiol”[Title/Abstract]OR“Vivelle”[Title/Abstract]OR“E_2_”[Title/Abstract]OR“Progesterone”[Mesh]OR“Pregnenedione”[Title/Abstract]OR“P”[Title/Abstract]OR“Testosterone”[Mesh]OR“Sustanon”[Title/Abstract]OR“8 Isotestosterone”[Title/Abstract]OR“Androderm”[Title/Abstract]OR“AndroGel”[Title/Abstract]OR“Andropatch”[Title/Abstract]OR“Androtop”[Title/Abstract]OR“Histerone”[Title/Abstract] OR“Sterotate”[Title/Abstract]OR“Testim”[Title/Abstract]OR“Testoderm”[Title/Abstract]OR“Testolin”[Title/Abstract]OR“Testopel”[Title/Abstract]OR“T”[Title/Abstract]OR“Prolactin”[Mesh]OR“Pituitary Lactogenic Hormone”[Title/Abstract]OR“Pituitary Mammotropic Hormone”[Title/Abstract]OR“Mammotropin”[Title/Abstract]OR“PRL”[Title/Abstract]OR“Anti-Mullerian Hormone” [Mesh]OR“Anti Mullerian Hormone”[Title/Abstract]OR“Mullerian Inhibiting”[Title/Abstract]OR“Mullerian Regression Factor”[Title/Abstract]OR“Mullerian Inhibitory Substance”[Title/Abstract]OR“Anti Muellerian Hormone”[Title/Abstract]OR“AMH”[Title/Abstract] #4“Randomized controlled trial”[Publication Type]OR“Randomized”[Title/Abstract]OR“controlled”[Title/Abstract]OR“Trial”[Title/Abstract] #5 #1 AND #2 AND #3 AND #4
Web of Science	#1 TS=(“Polycystic Ovary Syndrome”OR“Polycystic Ovarian Syndrome”OR“Sclerocystic Ovarian Degeneration”OR“Stein Leventhal Syndrome”OR“Sclerocystic Ovaries”OR“Ovary, Sclerocystic”OR“Sclerocystic Ovary”OR“PCOS”) #2 TS=(“Exercise”OR“Exercise,Physical”OR“Physical Exercise”OR“Exercise,Aerobic”OR“Aerobic Exercise”OR“Exercise, Isometric”OR“Isometric Exercise”OR“Acute Exercise”OR“Exercise, Acute”OR“Exercise Training”OR“Training,Exercise”OR“Physical Activity”OR“Activities, Physical”OR“Activity,Physical”OR“Physical Activities”OR“Running”OR“Jogging” OR“Fitness”OR“Yoga”OR“Exergaming”OR“Fit aerobics”OR“HIIT”OR“High-Intensity Interval Training”OR“Swim”OR“Walking”OR“Hiking”OR“Stair Climbing”OR“Racquet Sports”OR“Badminton”OR“Tennis”OR“Dancing”OR“Qigong”OR“Tai Ji”OR“Contrology”OR“cycling”OR“Strength”OR“Resistance”) #3 TS=(“Body Mass Index”OR“Quetelet Index”OR“BMI”OR“Follicle Stimulating Hormone”OR“Follitropin”OR“FSH”OR“Luteinizing Hormone”OR“Hormone, Luteinizing”OR“ICSH”OR“Interstitial Cell Stimulating Hormone”OR“Hormone, Interstitial Cell-Stimulating”OR“Lutropin”OR“Luteozyman”OR“Luteoziman”OR“LH”OR“Estradiol”OR“Oestradiol”OR“Estraderm TTS”OR“Estrace”OR“Ovocyclin”OR“Progynova”OR“Progynon Depot”OR“Delestrogen”OR“Aerodiol”OR“Vivelle”OR“E_2_”OR“Progesterone”OR“Pregnenedione”OR“P”OR“Testosterone”OR“Sustanon”OR“8 Isotestosterone”OR“Androderm”OR“AndroGel”OR“Andropatch”OR“Androtop”OR“Histerone”OR“Sterotate”OR“Testim”OR“Testoderm”OR“Testolin”OR“Testopel”OR“T”OR“Prolactin”OR“Pituitary Lactogenic Hormone”OR“Pituitary Mammotropic Hormone”OR“Mammotropin”OR“PRL”OR“Anti-Mullerian Hormone”OR“Anti Mullerian Hormone”OR“Mullerian Inhibiting”OR“Mullerian Regression Factor”OR“Mullerian Inhibitory Substance”OR“Anti Muellerian Hormone”OR“AMH”) #4 TS=(“Randomized controlled trial”OR“Randomized”OR“Controlled”OR“Trial”) #5 #1 AND #2 AND #3 AND #4
Cochrane Library	#1 Title Abstract Keyword=(“Polycystic Ovary Syndrome”OR“Polycystic Ovarian Syndrome”OR“Sclerocystic Ovarian Degeneration”OR“Stein Leventhal Syndrome”OR“Sclerocystic Ovaries”OR“Ovary, Sclerocystic”OR“Sclerocystic Ovary”OR“PCOS”) #2 Title Abstract Keyword=(“Exercise”OR“Exercise,Physical”OR“Physical Exercise”OR“Exercise,Aerobic”OR“Aerobic Exercise”OR“Exercise, Isometric”OR“Isometric Exercise”OR“Acute Exercise”OR“Exercise, Acute”OR“Exercise Training”OR“Training,Exercise”OR“Physical Activity”OR“Activities, Physical”OR“Activity,Physical”OR“Physical Activities”OR“Running”OR“Jogging” OR“Fitness”OR“Yoga”OR“Exergaming”OR“Fit aerobics”OR“HIIT”OR“High-Intensity Interval Training”OR“Swim”OR“Walking”OR“Hiking”OR“Stair Climbing”OR“Racquet Sports”OR“Badminton”OR“Tennis”OR“Dancing”OR“Qigong”OR“Tai Ji”OR“Contrology”OR“cycling”OR“Strength”OR“Resistance”) #3 Title Abstract Keyword=(“Body Mass Index”OR“Quetelet Index”OR“BMI”OR“Follicle Stimulating Hormone”OR“Follitropin”OR“FSH”OR“Luteinizing Hormone”OR“Hormone, Luteinizing”OR“ICSH”OR“Interstitial Cell Stimulating Hormone”OR“Hormone, Interstitial Cell-Stimulating”OR“Lutropin”OR“Luteozyman”OR“Luteoziman”OR“LH”OR“Estradiol”OR“Oestradiol”OR“Estraderm TTS”OR“Estrace”OR“Ovocyclin”OR“Progynova”OR“Progynon Depot”OR“Delestrogen”OR“Aerodiol”OR“Vivelle”OR“E_2_”OR“Progesterone”OR“Pregnenedione”OR“P”OR“Testosterone”OR“Sustanon”OR“8 Isotestosterone”OR“Androderm”OR“AndroGel”OR“Andropatch”OR“Androtop”OR“Histerone”OR“Sterotate”OR“Testim”OR“Testoderm”OR“Testolin”OR“Testopel”OR“T”OR“Prolactin”OR“Pituitary Lactogenic Hormone”OR“Pituitary Mammotropic Hormone”OR“Mammotropin”OR“PRL”OR“Anti-Mullerian Hormone”OR“Anti Mullerian Hormone”OR“Mullerian Inhibiting”OR“Mullerian Regression Factor”OR“Mullerian Inhibitory Substance”OR“Anti Muellerian Hormone”OR“AMH”) #4 Publication Type=(“Randomized controlled trial”)OR Title Abstract Keyword=(“Randomized”OR“controlled”OR“trial”) #5 #1 AND #2 AND #3 AND #4
Embase	#1“Ovary Polycystic Disease”[exp]OR“Polycystic Ovarian Syndrome” [ab,ti]OR“Sclerocystic Ovarian Degeneration”[ab,ti]OR“Stein Leventhal Syndrome”[ab,ti]OR“Sclerocystic Ovaries”[ab,ti]OR“Ovary, Sclerocystic”[ab,ti]OR“Sclerocystic Ovary”[ab,ti]OR“PCOS”[ab,ti] #2““Exercise”[exp]OR“Exercise,Physical”[ab,ti]OR“Physical Exercise”[ab,ti]OR“Exercise,Aerobic”[ab,ti]OR“Aerobic Exercise”[ab,ti]OR“Exercise, Isometric”[ab,ti]OR“Isometric Exercise”[ab,ti]OR“Acute Exercise”[ab,ti]OR“Exercise, Acute”[ab,ti]OR“Exercise Training”[ab,ti]OR“Training,Exercise”[ab,ti]OR“Physical Activity”[ab,ti]OR“Activities, Physical”[ab,ti]OR“Activity,Physical”[ab,ti]OR“Physical Activities”[ab,ti]OR“Running”[ab,ti]OR“Jogging”[ab,ti]OR“Fitness”[ab,ti]OR“Yoga”[ab,ti]OR“Exergaming”[ab,ti] OR“Fit aerobics”[ab,ti]OR“HIIT”[ab,ti]OR“High-Intensity Interval Training”[ab,ti]OR“Swim”[ab,ti]OR“Walking”[ab,ti]OR“Hiking”[ab,ti]OR“Stair Climbing”[ab,ti]OR“Racquet Sports”[ab,ti]OR“Badminton”[ab,ti]OR“Tennis”[ab,ti]OR“Dancing”[ab,ti]OR“Qigong”[ab,ti]OR“Tai Ji”[ab,ti]OR“Contrology”[ab,ti]OR“cycling”[ab,ti]OR“Strength”[ab,ti]OR“Resistance”[ab,ti] #3 “Body Mass”[exp]OR“Quetelet Index”[ab,ti]OR“BMI”[ab,ti]OR“Follitropin”[exp]OR“Follicle Stimulating Hormone”[ab,ti]OR“FSH”[ab,ti]OR“Luteinizing Hormone”[exp]OR“Hormone, Luteinizing”[ab,ti]OR“ICSH”[ab,ti]OR“Interstitial Cell Stimulating Hormone”[ab,ti]OR“Hormone, Interstitial Cell-Stimulating”[ab,ti]OR“Lutropin”[ab,ti]OR“Luteozyman”[ab,ti]OR“Luteoziman”[ab,ti]OR“LH”[ab,ti]OR“Estradiol”[exp]OR“Oestradiol”[ab,ti]OR“Estraderm TTS”[ab,ti]OR“Estrace”[ab,ti]OR“Ovocyclin”[ab,ti]OR“Progynova”[ab,ti]OR“Progynon Depot”[ab,ti]OR“Delestrogen”[ab,ti]OR“Aerodiol”[ab,ti]OR“Vivelle”[ab,ti]OR“E_2_”[ab,ti]OR“Progesterone”[exp]OR“Pregnenedione”[ab,ti]OR“P”[ab,ti]OR“Testosterone”[exp]OR“Sustanon”[ab,ti]OR“8 Isotestosterone”[ab,ti]OR“Androderm”[ab,ti]OR“AndroGel”[ab,ti]OR“Andropatch”[ab,ti]OR“Androtop”[ab,ti]OR“Histerone”[ab,ti]OR“Sterotate”[ab,ti]OR“Testim”[ab,ti]OR“Testoderm”[ab,ti]OR“Testolin”[ab,ti]OR“Testopel”[ab,ti]OR“T”[ab,ti]OR“Prolactin”[exp]OR“Pituitary Lactogenic Hormone”[ab,ti]OR“Pituitary Mammotropic Hormone”[ab,ti]OR“Mammotropin”[ab,ti]OR“PRL”[ab,ti]OR“Muellerian inhibiting factor”[exp]OR“Anti Mullerian Hormone”[ab,ti]OR“Mullerian Regression Factor”[ab,ti]OR“Mullerian Inhibitory Substance”[ab,ti]OR“Anti Muellerian Hormone”[ab,ti]OR“AMH”[ab,ti] #4“Randomized controlled trial”[exp]OR“Randomized”[ab,ti]OR“Controlled”[ab,ti]OR“Trial”[ab,ti] #5 #1 AND #2 AND #3 AND #4

This study was independently conducted by XC and DC, utilizing a search strategy that strictly adhered to the Cochrane Handbook guidelines. The search process and results were reported in compliance with the Preferred Reporting Items for Systematic Reviews and Meta-Analyses (PRISMA) guidelines (Cumpston et al., 2019). Disagreements were resolved through discussion or consultation with a third reviewer to reach consensus on study eligibility. Notably, no major discrepancies occurred during the search process. This study was registered in the PROSPERO database under registration number CRD420251108464.

### Literature screening and data

The retrieved literature was imported into EndNote 20 for deduplication. Two reviewers (XC and DC) independently screened the records based on the inclusion and exclusion criteria, extracted the data, and cross-checked the results. Disagreements were resolved through discussion or by consulting a third reviewer to reach a consensus. Notably, no significant disagreements were encountered during the data extraction process. The data extraction form had been designed and piloted in advance, with no subsequent modifications required.

Extracted information encompassed: (1) study country, first author, publication year, and study design; (2) participant characteristics, including group sample sizes, mean BMI, and average age; (3) core parameters of the exercise intervention, including modality, intensity, and intervention duration; and (4) outcome indicators and blood sampling times.

### Quality assessment of included studies

The methodological quality of randomized controlled trials (RCTs) included in this review was evaluated using the PEDro scale (Physiotherapy Evidence Database) (Verhagen et al., 1998). This scale encompasses ten criteria: random allocation, allocation concealment, baseline comparability, participant blinding, therapist blinding, assessor blinding, retention rate exceeding 85%, intention-to-treat analysis, between-group statistical comparisons, and presentation of point estimates along with measures of variability. Each criterion met is scored as one point; failure to meet a criterion scores zero. Total scores range from 0 to 10, with scores below 4 considered poor quality, 4–5 moderate quality, 6–8 good quality, and 9–10 high quality. Only studies with scores above 4 were included in the analysis.

The overall quality of evidence was appraised using GRADE profiler software, evaluating five downgrading domains: publication bias, inconsistency, imprecision, indirectness, and study limitations (Guyatt et al., 2008). Based on the severity of downgrades, evidence quality was categorized into four levels: high (no downgrades), moderate (downgraded by one level), low (downgraded by two levels), and very low (downgraded by three levels). Scoring was carried out independently by two authors, with two copies generated. In cases of disagreement, a third researcher participated in the adjudication process, and discussions continued until a consensus was achieved.

### Statistical methods

To account for the non-independence of effect sizes, we performed a three-level hierarchical meta-analysis using the metafor package in R, employing a random-effects framework (Cui et al., 2024; Pustejovsky & Tipton, 2022). Models were estimated *via* restricted maximum likelihood (REML), with Hedges’ g and 95% confidence intervals (CI) serving as the metric for effect size (Riley, Higgins & Deeks, 2011). Analyses were restricted to outcomes with ${\rm k} > 10$ effect sizes to ensure statistical viability (Nakagawa et al., 2017). Global heterogeneity was evaluated using Cochran’s Q test (Cochran, 1954), while ${{\rm I}^2}$ statistics partitioned variance across Level 1 (sampling error), Level 2 (within-study), and Level 3 (between-study) components (Assink & Wibbelink, 2024). The stability of the findings was scrutinized through influence diagnostics and leave-one-out sensitivity analyses (Riley, Higgins & Deeks, 2011). To justify the hierarchical structure, likelihood ratio tests (LRT) compared the goodness-of-fit between three-level and traditional two-level model (Cheung, 2014). Publication bias was assessed *via* Egger’s regression (Egger et al., 1997), supplemented by the trim-and-fill method where necessary (Duval & Tweedie, 2000). Potential sources of heterogeneity were explored through subgroup analyses based on exercise modality, intensity, intervention duration, mean BMI, mean age, and blood sampling timing (specifically, the early follicular phase, days 2–5).

This part was primarily spearheaded by YL. His work included executing the three-level meta-analysis using R software, which involved sophisticated code development, model fitting, and heterogeneity testing. Furthermore, he performed moderator analyses and created professional data visualizations to significantly enhance the clarity and interpretability of the results.

## Results

### Literature screening and inclusion

**Figure 1:**
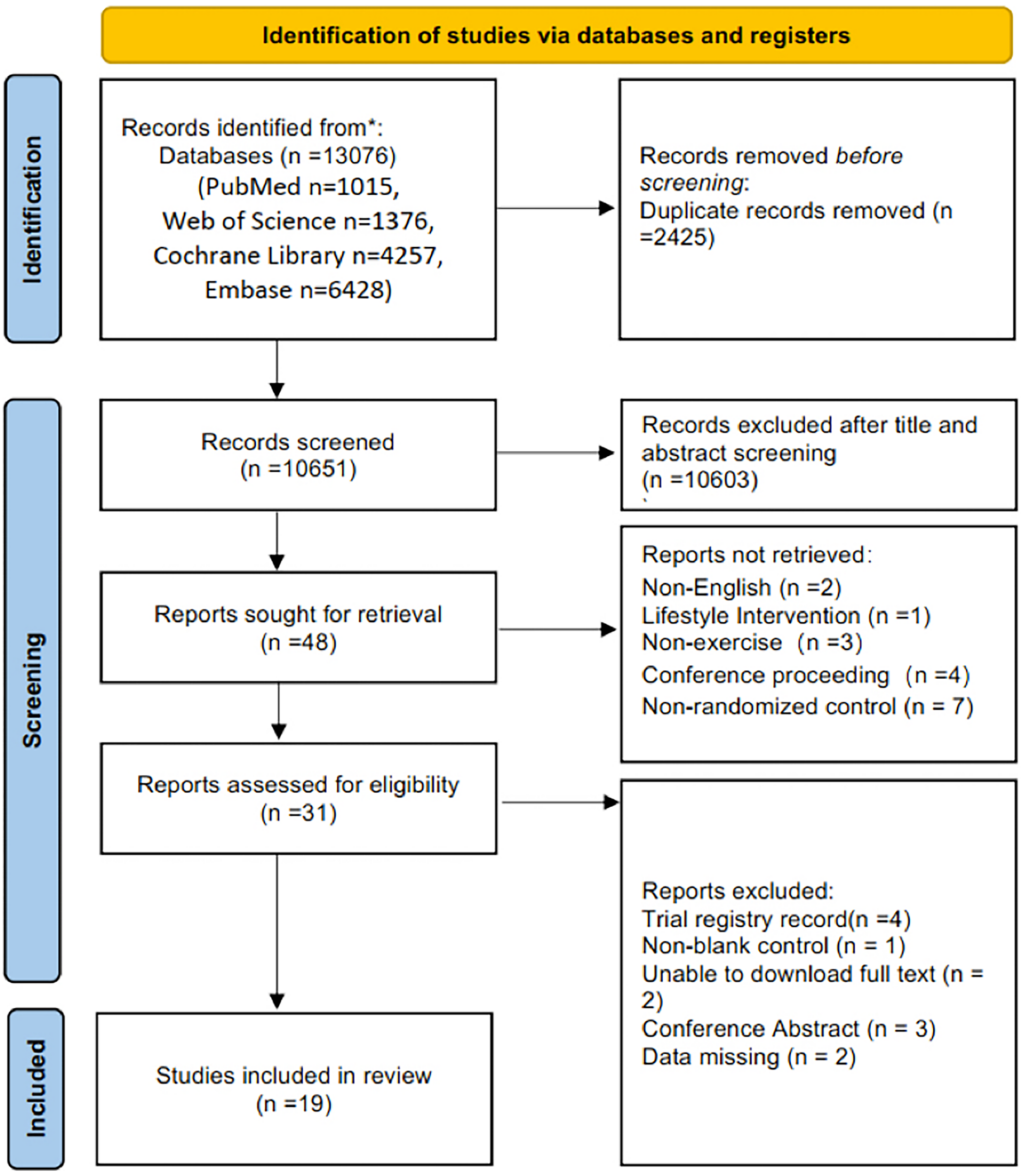
Literature screening flowchart.

A total of 13,076 articles were retrieved, including 1,015 from PubMed, 1,376 from Web of Science, 4,257 from Cochrane Library, and 6,428 from Embase. After deduplication, 10,651 articles remained. Titles and abstracts were reviewed to exclude irrelevant articles (*e.g*., reviews, conference articles, commentaries, animal studies), reducing the number to 48. Further screening excluded studies based on criteria such as non-English language, academic essays, lifestyle interventions, medication studies, inconsistent outcome measures, non-blank controls, study protocols, inaccessible full texts, inability to extract results, non-randomized controls, missing data, and questionnaire-based studies. Finally, 19 articles were included for analysis (Almenning et al., 2015; Benham et al., 2021; Costa et al., 2018; Giallauria et al., 2008; Jedel et al., 2011; Kiel et al., 2022; Kumari et al., 2025; Lopes et al., 2018; Mohammadi, Monazzami & Alavimilani, 2023; Nasiri et al., 2025; Philbois et al., 2022; Ribeiro et al., 2020, 2021a, 2021b; Sverrisdottir et al., 2009; Turan et al., 2015; Vigorito et al., 2007; Vizza et al., 2016; Wu et al., 2021) (Fig. 1).

### Characteristics of included studies

In the 19 articles included, there are 11 studies with parallel two-group designs and eight studies with parallel three-group designs, involving a total of 1,018 participants (595 in the experimental group and 423 in the control group), all of whom are adult women aged between 18 and 45 years. For the studies with parallel three-group designs, due to the inclusion of multiple intervention groups, this study employed an equal allocation method for the control group sample size to prevent double counting of participants. For articles that do not clearly report exercise intensity, the intensity will be defined based on the stated intensity in accordance with relevant guidelines (Borg, 1970; American College of Sports Medicine, 2014) (Table 2).

**Table 2:** Characteristics of included studies.

Country where the study was conducted, first author, year of publication	Study design	Duration of intervention	Intensity of exercise	Metabolic category/type	Intervention group			Control group			Timing of blood sampling	Outcome measures
					*n*	BMI	Age	*n*	BMI	Age		
Italy, Vigorito et al. (2007)	Two-arm parallel	12 weeks	Moderate	Aerobic	45	29.3 ± 2.9	21.7 ± 2.3	45	29.4 ± 3.5	21.9 ± 1.9	Early follicular phase	BMI, FSH, LH, PRL, E2, P, T
Italy, Giallauria et al. (2008)	Two-arm parallel	12 weeks	Moderate	Aerobic	62	29.2 ± 2.9	22.8 ± 3.7	62	29.5 ± 3.5	22.6 ± 3.0	Early follicular phase	BMI, FSH, LH, PRL, E2, P
Sweden, Sverrisdottir et al. (2009)	Two-arm parallel	16 weeks	Moderate	Aerobic	5	26.8 ± 4.8	30.4 ± 5.5	6	28.0 ± 6.2	31.0 ± 3.2	Not reported	BMI, FSH, LH, T
Sweden, Jedel et al. (2011)	Two-arm parallel	16 weeks	Moderate	Aerobic	30	27.7 ± 6.4	30.2 ± 4.7	15	26.8 ± 5.56	30.1 ± 4.2	Not reported	BMI, FSH, LH, E2, T
Norway, Almenning et al. (2015)①	Three-arm parallel	10 weeks	High	Mixed	8	27.4 ± 6.9	Not reported	4	26.5 ± 5.0	Not reported	Menstruation	BMI, AMH
Norway, Almenning et al. (2015)②	Three-arm parallel	10 weeks	Moderate	Anaerobic	8	26.1 ± 6.5	Not reported	5	26.5 ± 5.0	Not reported	Menstruation	BMI, AMH
Turkey, Turan et al. (2015)	Two-arm parallel	8 weeks	Moderate	Mixed	14	21.8 ± 1.0	Not reported	16	21.9 ± 1.1	Not reported	Early follicular phase	BMI, FSH, LH, E2, T
Australia, Vizza et al. (2016)	Two-arm parallel	12 weeks	Moderate	Anaerobic	7	41.3 ± 12.5	26 ± 7	6	34.0 ± 9.4	29 ± 3	Not reported	BMI, T
Brazil, Costa et al. (2018)	Two-arm parallel	16 weeks	High	Aerobic	14	32 ± 4.2	27.6 ± 4.5	13	33.6 ± 5.1	24.4 ± 5.0	Not reported	BMI
Brazil, Lopes et al. (2018)①	Three-arm parallel	16 weeks	Moderate	Aerobic	23	29.3 ± 5.6	30.2 ± 5.1	12	29.9 ± 5.3	28.8 ± 6.0	Early follicular phase	BMI, T
Brazil, Lopes et al. (2018)②	Three-arm parallel	16 weeks	High	Aerobic	22	29.0 ± 4.8	29.4 ± 4.1	12	29.9 ± 5.3	28.8 ± 6.0	Early follicular phase	BMI, T
Brazil, Ribeiro et al. (2021a)①	Three-arm parallel	16 weeks	Moderate	Aerobic	28	28.4 ± 5.6	29.1 ± 5.3	15	29.1 ± 5.2	28.5 ± 5.8	Early follicular phase	BMI, T
Brazil, Ribeiro et al. (2021a)②	Three-arm parallel	16 weeks	High	Aerobic	29	28.7 ± 4.8	29 ± 4.3	15	29.1 ± 5.2	28.5 ± 5.8	Early follicular phase	BMI, T
Brazil, Ribeiro et al. (2020)①	Three-arm parallel	16 weeks	Moderate	Aerobic	28	28.4 ± 5.6	29.1 ± 5.3	15	29.1 ± 5.2	28.5 ± 5.8	Early follicular phase	BMI, FSH, LH, E2, T
Brazil, Ribeiro et al. (2020)②	Three-arm parallel	16 weeks	High	Aerobic	29	28.7 ± 4.8	29 ± 4.3	15	29.1 ± 5.2	28.5 ± 5.8	Early follicular phase	BMI, FSH, LH, E2, T
China, Wu et al. (2021)	Two-arm parallel	12 weeks	Moderate	Aerobic	19	23.8 ± 3.0	32.7 ± 3.2	19	24.1 ± 3.2	33.2 ± 2.9	Early follicular phase	BMI, FSH, AMH
Brazil, Ribeiro et al. (2021b)①	Three-arm parallel	16 weeks	Moderate	Aerobic	28	28.43 ± 5.62	29.14 ± 5.26	15	29.09 ± 5.25	28.5 ± 5.76	Not reported	BMI, FSH, LH, E2, T
Brazil, Ribeiro et al. (2021b)②	Three-arm parallel	16 weeks	High	Aerobic	29	28.67 ± 4.76	28.97 ± 4.32	15	29.09 ± 5.25	28.5 ± 5.76	Not reported	BMI, FSH, LH, E2, T
Canada, Benham et al. (2021)①	Three-arm parallel	24 weeks	High	Mixed	11	31.4 ± 8.6	29.1 ± 4.1	7	31.6 ± 8.2	29.1 ± 5.4	Not reported	BMI
Canada, Benham et al. (2021)②	Three-arm parallel	24 weeks	Moderate	Aerobic	12	31.3 ± 9.0	29.5 ± 4.6	8	31.6 ± 8.2	29.1 ± 5.4	Not reported	BMI
Norway, Kiel et al. (2022)①	Three-arm parallel	16 weeks	High	Mixed	16	30.8 ± 7.2	30.1 ± 4.9	10	31.2 ± 6.7	28.3 ± 5.3	Not reported	BMI, T, AMH
Norway, Kiel et al. (2022)②	Three-arm parallel	16 weeks	Moderate	Mixed	17	29.5 ± 5.7	30.4 ± 5.0	10	31.2 ± 6.7	28.3 ± 5.3	Not reported	BMI, T, AMH
Brazil, Philbois et al. (2022)①	Three-arm parallel	16 weeks	Moderate	Aerobic	25	27.7 ± 5.7	29 ± 5	12	31.2 ± 6.7	29 ± 5	Early follicular phase	BMI, T
Brazil, Philbois et al. (2022)②	Three-arm parallel	16 weeks	High	Mixed	25	27.8 ± 4.2	29 ± 4	13	29.2 ± 5.4	29 ± 5	Early follicular phase	BMI, T
Iran, Mohammadi, Monazzami & Alavimilani (2023)	Two-arm parallel	8 weeks	High	Mixed	14	29.5 ± 4.5	24.2 ± 4.8	14	31.4 ± 2.6	22.9 ± 5.3	Not reported	BMI
Iran, Nasiri et al. (2025)	Two-arm parallel	8 weeks	Moderate	Mixed	15	30.7 ± 3.7	24.4 ± 5.5	15	29.9 ± 3.4	23.1 ± 5.1	Not reported	AMH, T
India, Kumari et al. (2025)	Two-arm parallel	12 weeks	Moderate	Aerobic	32	25.728 ± 5.586	23 ± 4.410	29	25.578 ± 4.779	23.900 ± 3.630	Not reported	BMI, FSH, LH, AMH, T

**Note:**

BMI, Body mass index; T, testosterone; FSH, follicle-stimulating hormone; LH, luteinizing hormone; E_2_, estradiol; AMH, anti-Müllerian hormone; PRL, prolactin; P, progesterone.

### Quality assessment of included studies

All included studies reported adequate randomization, baseline comparability, and provided point estimates with measures of variability. Allocation concealment was reported in 15 studies; 10 studies achieved retention rates above 85%; eight studies reported between-group statistical comparisons and intention-to-treat analyses; three studies implemented blinded outcome assessment; none reported participant or therapist blinding. PEDro scores ranged from 4 to 8, with nine studies scoring 6–8 indicating good quality, and 10 scoring 4–5 indicating moderate quality. The mean PEDro score was 5.3, suggesting overall moderate methodological quality (Table 3).

**Table 3:** Methodological quality assessment of included studies.

Included studies	Randomized allocation	Allocation concealment	Similarity of baseline characteristics	Blinding of study subjects	Blinding of therapists	Blinding of outcome assessors	Participation rate above 85%	Intention-to-treat analysis	Between-group statistical analysis	Point measurement and Difference score	Overall score
Italy, Vigorito et al. (2007)	1	0	1	0	0	1	1	1	0	1	6
Italy, Giallauria et al. (2008)	1	0	1	0	0	1	1	1	0	1	6
Sweden, Sverrisdottir et al. (2009)	1	1	1	0	0	1	0	0	1	1	6
Sweden, Jedel et al. (2011)	1	1	1	0	0	0	0	1	1	1	6
Norway, Almenning et al. (2015)	1	1	1	0	0	0	0	0	1	1	5
Turkey, Turan et al. (2015)	1	0	1	0	0	0	1	1	0	1	5
Australia, Vizza et al. (2016)	1	1	1	0	0	0	1	0	1	1	6
Brazil, Costa et al. (2018)	1	1	1	0	0	0	0	0	1	1	5
Brazil, Lopes et al. (2018)	1	1	1	0	0	0	0	0	0	1	4
Brazil, Ribeiro et al. (2021a)	1	1	1	0	0	0	0	0	0	1	4
Brazil, Ribeiro et al. (2020)	1	1	1	0	0	0	0	0	0	1	4
China, Wu et al. (2021)	1	0	1	0	0	0	1	1	1	1	6
Brazil, Ribeiro et al. (2021b)	1	1	1	0	0	0	0	0	0	1	4
Canada, Benham et al. (2021)	1	1	1	0	0	0	1	1	0	1	6
Norway, Kiel et al. (2022)	1	1	1	0	0	0	1	0	0	1	5
Brazil, Philbois et al. (2022)	1	1	1	0	0	0	1	1	1	1	7
Iran, Mohammadi, Monazzami & Alavimilani (2023)	1	1	1	0	0	0	1	0	0	1	5
Iran, Nasiri et al. (2025)	1	1	1	0	0	0	1	1	0	1	6
India, Kumari et al. (2025)	1	1	1	0	0	0	0	0	1	1	5

### Meta-analysis results

#### Effects of exercise on reproductive endocrine hormones in adult women with PCOS

**Figure 2:**
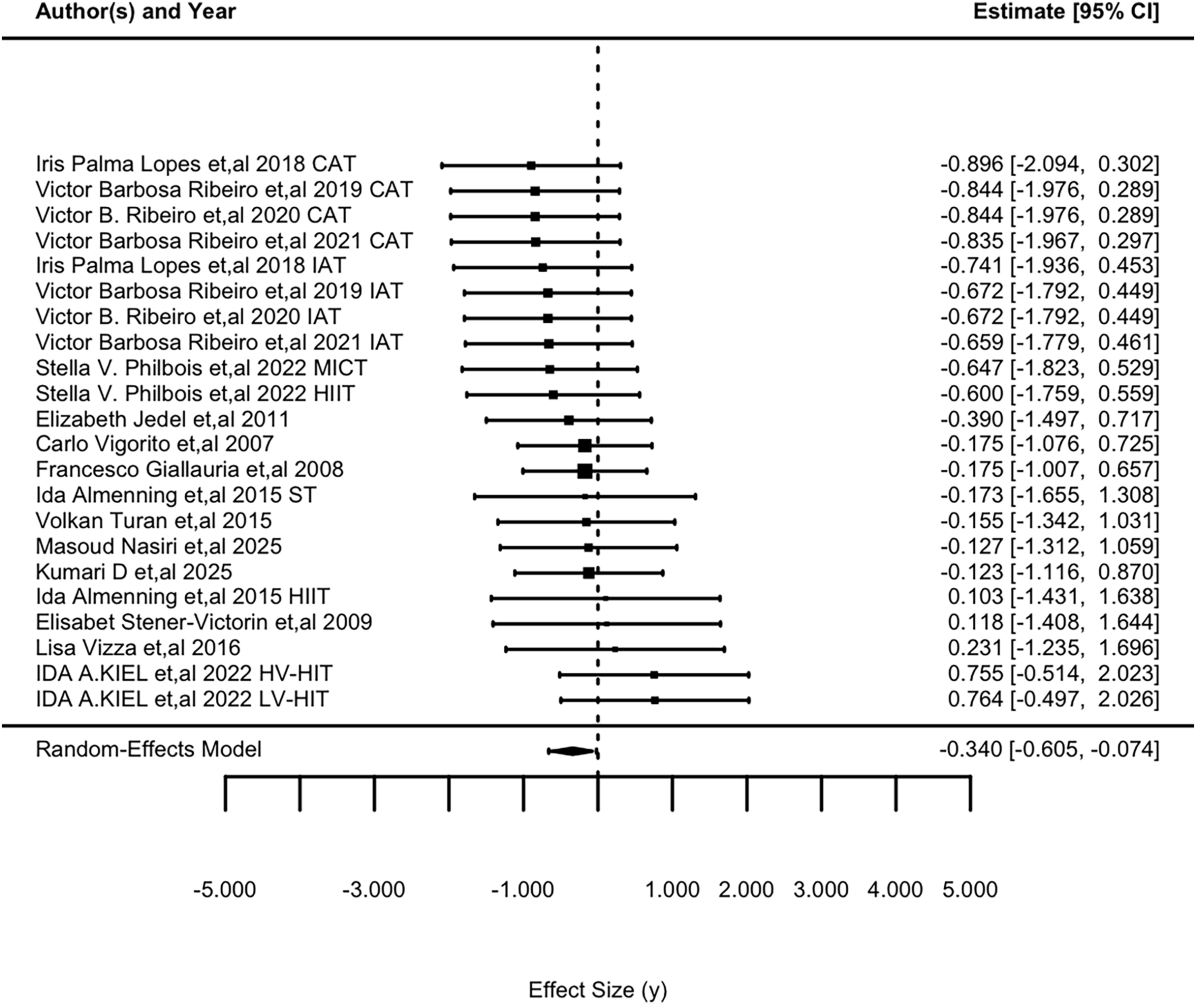
Forest plot for T.

The impact of exercise on T levels was synthesized from 15 studies ( ${\rm k} = 22$) encompassing 887 participants (Fig. 2). Both three-level and two-level meta-analyses yielded identical, significant overall effects (g = −0.3396, 95% CI [−0.6050 to −0.0741], *P* = 0.0146). Global heterogeneity was negligible, Q(df = 21) = 12.7492, *P* = 0.9172, with variance partitioning revealing that sampling error (Level 1) accounted for 97.8760% of the total variance. Neither within-study (Level 2: 0%) nor between-study (Level 3: 2.1240%) variance components reached statistical significance. Likelihood ratio tests (LRT) confirmed that the inclusion of within-study and between-study variance components did not significantly improve model fit (LRT = 0.0000, *P* = 0.5000; and LRT = 0.0087, *P* = 0.4628, respectively).

**Figure 3:**
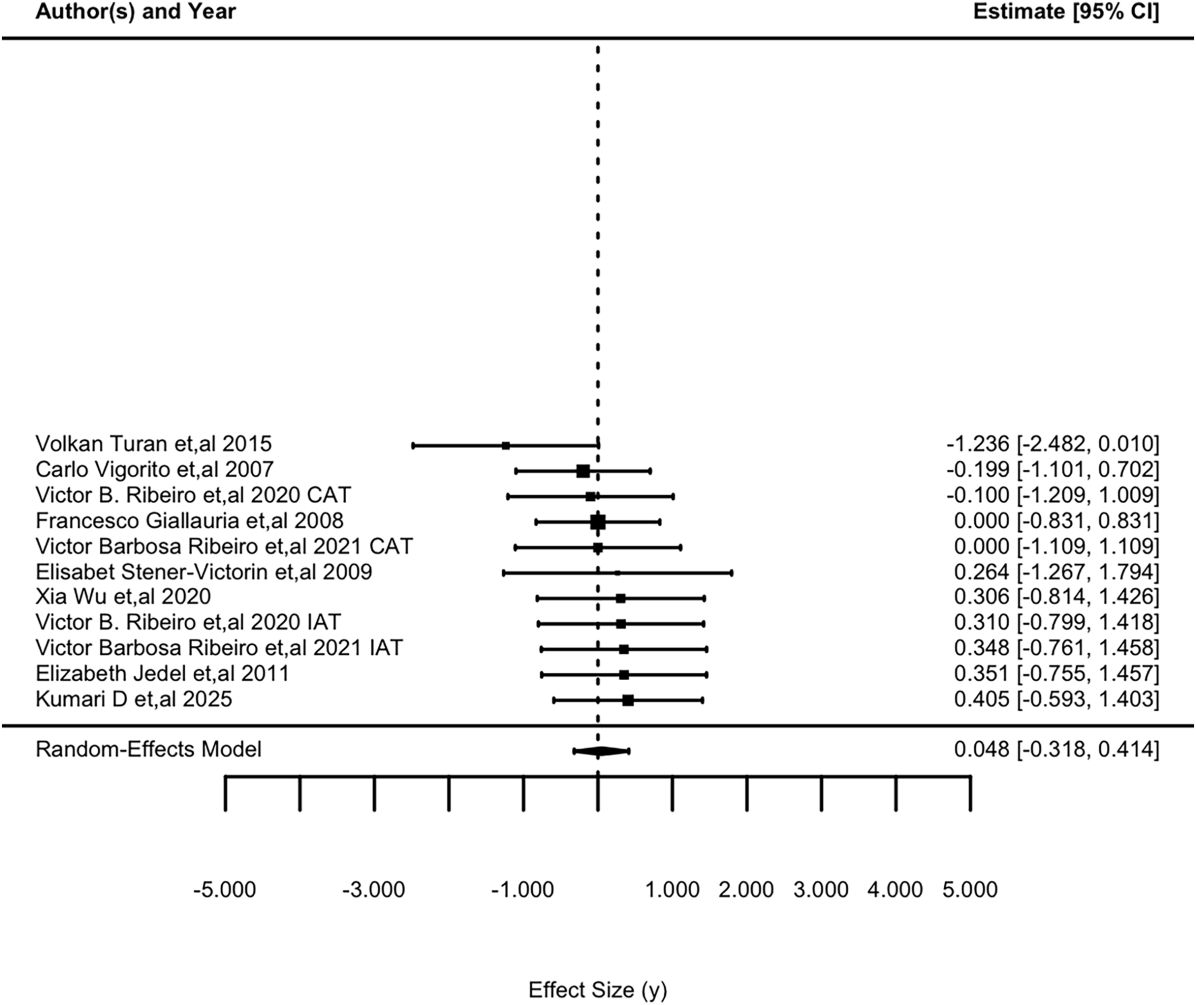
Forest plot for FSH.

The impact of exercise on FSH levels was synthesized from nine studies ( ${\rm k} = 11$) encompassing 573 participants (Fig. 3). Both three-level and two-level meta-analyses yielded identical, non-significant overall effects (g = 0.0480, 95% CI [−0.3185 to 0.4145], *P* = 0.7764). Global heterogeneity was negligible, Q(df = 10) = 6.0155, *P* = 0.8140, with variance partitioning revealing that sampling error (Level 1) accounted for 100% of the total variance. Neither within-study (Level 2: 0%) nor between-study (Level 3: 0%) variance components reached statistical significance. Likelihood ratio tests (LRT) confirmed that the inclusion of within-study and between-study variance components did not significantly improve model fit (LRT = 0.0000, *P* = 0.5000; and LRT = 0.0087, *P* = 0.5000, respectively).

The effects of exercise on other endocrine markers were also examined, including LH (eight studies, ${\rm k} = 10$), ${{\rm E}_2}$ (six studies, ${\rm k} = 8$), AMH (five studies, ${\rm k} = 7$), *P* (two studies, ${\rm k} = 2$), and PRL (two studies, ${\rm k} = 2$). However, these outcomes did not satisfy the requirements for three-level meta-analysis, precluding a quantitative synthesis for these variables.

#### The impact of exercise on body mass index in adult patients with PCOS

**Figure 4:**
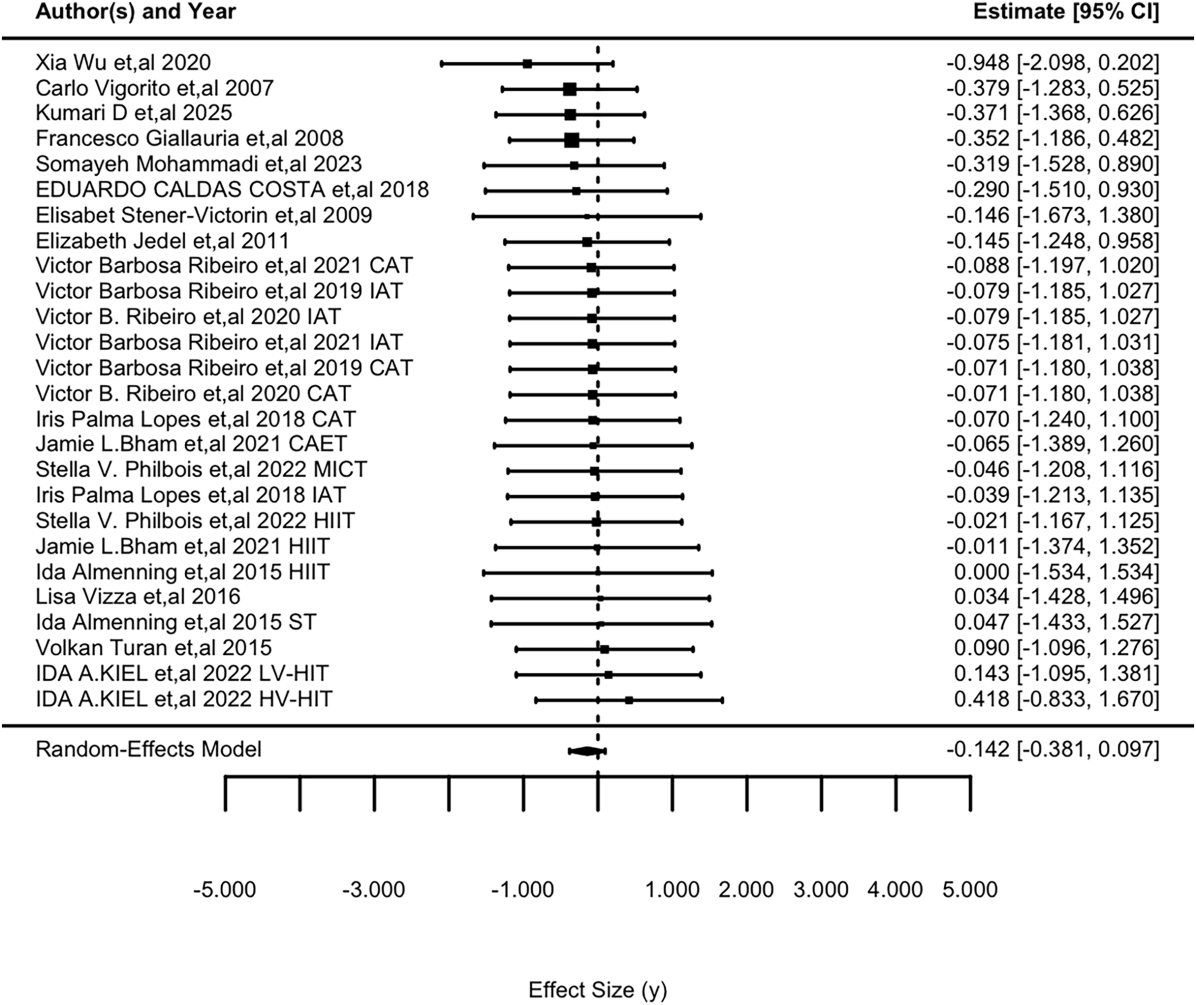
Forest plot for BMI.

The impact of exercise on BMI levels was synthesized from 18 studies (k = 26) encompassing 988 participants (Fig. 4). Both three-level and two-level meta-analyses yielded identical, non-significant overall effects (g = −0.1419, 95% CI [−0.3808 to 0.0971], *P* = 0.2328). Global heterogeneity was negligible, Q(df = 25) = 4.2483, *P* = 1.0000, with variance partitioning revealing that sampling error (Level 1) accounted for 100% of the total variance. Neither within-study (Level 2: 0%) nor between-study (Level 3: 0%) variance components reached statistical significance. Likelihood ratio tests (LRT) confirmed that the inclusion of within-study and between-study variance components did not significantly improve model fit (LRT = 0.0000, *P* = 0.5000; and LRT = 0.0087, *P* = 0.5000, respectively).

### Subgroup analysis

To explore potential sources of heterogeneity, subgroup analyses were stratified by exercise modality, intensity, intervention duration, mean BMI, and mean age. Additionally, the timing of blood sampling—specifically whether it occurred during the early follicular phase (days 2–5 of the menstrual cycle)—was also evaluated as a categorical moderator.

#### Subgroup analysis of exercise effects on T

Subgroup analysis revealed no significant differences between exercise modalities (F = 2.2781, *P* = 0.1298). The “aerobic metabolism” group reached statistical significance (k = 14, g = −0.5120, 95% CI [−0.8230 to −0.2010], *P* = 0.0027). In contrast, the “anaerobic metabolism” and “mixed metabolism” groups did not show any statistical significance. There were no inter-group differences in exercise intensity (F = 0.1524, *P* = 0.7004). Neither the “high-intensity” group nor the “moderate-intensity” group was statistically significant. Moderator analysis indicated no significant inter-group differences based on mean BMI (F = 3.1360, *P* = 0.0665). The “25 to 29.9” group showed statistical significance (k = 17, g = −0.4859, 95% CI [−0.7775 to −0.1943], *P* = 0.0025). However, the “18 to 24.9” and “30 or greater” groups were not statistically significant. No significant between-group differences were observed when stratified by mean age (F = 0.5837, *P* = 0.6334). The “26 to 30 years old” group was statistically significant (k = 13, g = −0.4878, 95% CI [−0.8465 to −0.1290], *P* = 0.0105). The groups for “21 to 25 years old,” “Over 30 years old,” and “Not reported” were not statistically significant. Regarding intervention duration and the timing of blood sampling, the mixed-effects models failed to reach convergence (convergence = 1). This suggests that the algorithm was unable to identify a local optimum within the pre-specified maximum number of iterations, thus precluding the completion of these subgroup analyses (Table 4).

**Table 4:** Summary of analytical results for T.

Moderator variable	K	g (95% CI)	*P*	F(df1, df2)
Exercise modalities	22			F(df1 = 2, df2 = 19) = 2.2781, *P* = 0.1298
Aerobic metabolism	14	−0.5120 [−0.8230 to −0.2010]	0.0027	
Anaerobic metabolism	2	0.0309 [−1.0818 to 1.1435]	0.9543	
Mixed metabolism	6	0.0901 [−0.4547 to 0.6350]	0.7330	
Training intensity	22			F(df1 = 1, df2 = 20) = 0.1524, *P* = 0.7004
High	7	−0.4169 [−0.9006 to 0.0668]	0.0873	
Moderate	15	−0.3093 [−0.6225 to 0.0038]	0.0526	
Duration of intervention	22	Optimizer (nlminb) did not achieve convergence (convergence = 1).		
More than 12 weeks	14			
12 weeks or less	8			
Mean BMI	22			F(df1 = 2, df2 = 19) = 3.1360, *P* = 0.0665
18 to 24.9	1	−0.1554 [−1.4227 to 1.1119]	0.8002	
25 to 29.9	17	−0.4859 [−0.7775 to −0.1943]	0.0025	
30 or greater	4	0.3982 [−0.2871 to 1.0836]	0.2388	
Mean age	22			F(df1 = 3, df2 = 18) = 0.5837, *P* = 0.6334
21 to 25 years old	4	−0.1551 [−0.6747 to 0.3645]	0.5385	
26 to 30 years old	13	−0.4878 [−0.8465 to −0.1290]	0.0105	
Over 30 years old	2	−0.2135 [−1.1840 to 0.7569]	0.6494	
Not reported	3	−0.0915 [−0.9517 to 0.7687]	0.8256	
Blood sampling time	22	Optimizer (nlminb) did not achieve convergence (convergence = 1).		
Is 2 to 5 days	11			
Is not 2 to 5 days	2			
Not reported	9			

**Note:**

K, Effect size quantity.

#### Subgroup analysis of exercise effects on FSH

Subgroup analyses revealed that the intervention effects were not significantly moderated by any of the categorical variables. Specifically, no significant between-group differences were observed for exercise modality (F = 4.3751, *P* = 0.0660), intensity (F = 0.5942, *P* = 0.4605), intervention duration (F = 0.6518, *P* = 0.4403), mean BMI (F = 1.2124, *P* = 0.2994), mean age (F = 1.5821, *P* = 0.2773), or blood sampling timing (F = 1.3539, *P* = 0.2745). Furthermore, within-group analysis indicated that none of the individual subgroups within these categories reached statistical significance (Table 5).

**Table 5:** Summary of analytical results for FSH.

Moderator variable	K	g (95% CI)	*P*	F(df1, df2)
Exercise modalities	11			F(df1 = 1, df2 = 9) = 4.3751, *P* = 0.0660
Aerobic metabolism	10	0.1401 [−0.2450 to 0.5253]	0.4318	
Anaerobic metabolism	0			
Mixed metabolism	1	−1.2364 [−2.6744 to 0.2016]	0.0836	
Training intensity	11			F(df1 = 1, df2 = 9) = 0.5942, *P* = 0.4605
High	2	0.3291 [−0.5759 to 1.2342]	0.4320	
Moderate	9	−0.0092 [−0.4173 to 0.3990]	0.9605	
Duration of intervention	11			F(df1 = 1, df2 = 9) = 0.6518, *P* = 0.4403
More than 12 weeks	6	0.1898 [−0.3545 to 0.7340]	0.4506	
12 weeks or less	5	−0.0764 [−0.5861 to 0.4334]	0.7424	
Mean BMI	11			F(df1 = 1, df2 = 9) = 1.2124, *P* = 0.2994
18 to 24.9	2	−0.3835 [−1.3449 to 0.5779]	0.3904	
25 to 29.9	9	0.1240 [−0.2795 to 0.5275]	0.5045	
30 or greater	0			
Mean age	11			F(df1 = 3, df2 = 7) = 1.5821, *P* = 0.2773
21 to 25 years old	3	0.0437 [−0.5847 to 0.6722]	0.8739	
26 to 30 years old	4	0.1396 [−0.5293 to 0.8086]	0.6368	
Over 30 years old	3	0.3150 [−0.5295 to 1.1594]	0.4070	
Not reported	1	−1.2364 [−2.7396 to 0.2668]	0.0929	
Blood sampling time	11			F(df1 = 1, df2 = 9) = 1.3539, *P* = 0.2745
Is 2 to 5 days	6	−0.1091 [−0.5905 to 0.3723]	0.6204	
Is not 2 to 5 days	0			
Not reported	5	0.2811 [−0.3053 to 0.8675]	0.3064	

**Note:**

K, Effect size quantity.

#### Subgroup analysis of exercise effects on BMI

Subgroup analyses indicated that the intervention effects were consistent across all examined moderators, with no significant between-group differences detected. Specifically, there were no significant differences based on exercise modality (F = 0.4753, *P* = 0.6277), intensity (F = 0.3031, *P* = 0.5870), intervention duration (F = 1.1457, *P* = 0.2951), mean BMI (F = 0.4862, *P* = 0.6211), or mean age (F = 0.7552, *P* = 0.5312). Furthermore, none of the individual subgroups within these categories—including varied metabolic types, BMI ranges, and age cohorts—yielded statistically significant results (Table 6).

**Table 6:** Summary of analytical results for BMI.

Moderator variable	K	g (95% CI)	*P*	F(df1, df2)
Exercise modalities	26			F(df1 = 2, df1 = 23) = 0.4753, *P* = 0.6277
Aerobic metabolism	17	−0.2117 [−0.4936 to 0.0703]	0.1342	
Anaerobic metabolism	2	0.0400 [−1.0577 to 1.1378]	0.9405	
Mixed metabolism	7	0.0417 [−0.4609 to 0.5444]	0.8651	
Training intensity	26			F(df1 = 1, df2 = 24) = 0.3031, *P* = 0.5870
High	10	−0.0561 [−0.4570 to 0.3449]	0.7754	
Moderate	16	−0.1894 [−0.4879 to 0.1091]	0.2027	
Duration of intervention	26			F(df1 = 1, df2 = 24) = 1.1457, *P* = 0.2951
More than 12 weeks	17	−0.0466 [−0.3484 to 0.2552]	0.7529	
12 weeks or less	9	−0.3037 [−0.6970 to 0.0896]	0.1241	
Mean BMI	26			F(df1 = 2, df2 = 23) = 0.4862, *P* = 0.6211
18 to 24.9	2	−0.4449 [−1.3163 to 0.4264]	0.3018	
25 to 29.9	18	−0.1555 [−0.4342 to 0.1233]	0.2604	
30 or greater	6	0.0390 [−0.5221 to 0.6001]	0.8870	
Mean age	26			F(df1 = 3, df2 = 22) = 0.7552, *P* = 0.5312
21 to 25 years old	4	−0.3587 [−0.8660 to 0.1485]	0.1566	
26 to 30 years old	16	−0.0324 [−0.3459 to 0.2811]	0.8321	
Over 30 years old	3	−0.4477 [−1.1945 to 0.2991]	0.2268	
Not reported	3	0.0536 [−0.7849 to 0.8921]	0.8957	

**Note:**

K, Effect size quantity.

### Influential analysis

**Figure 5:**
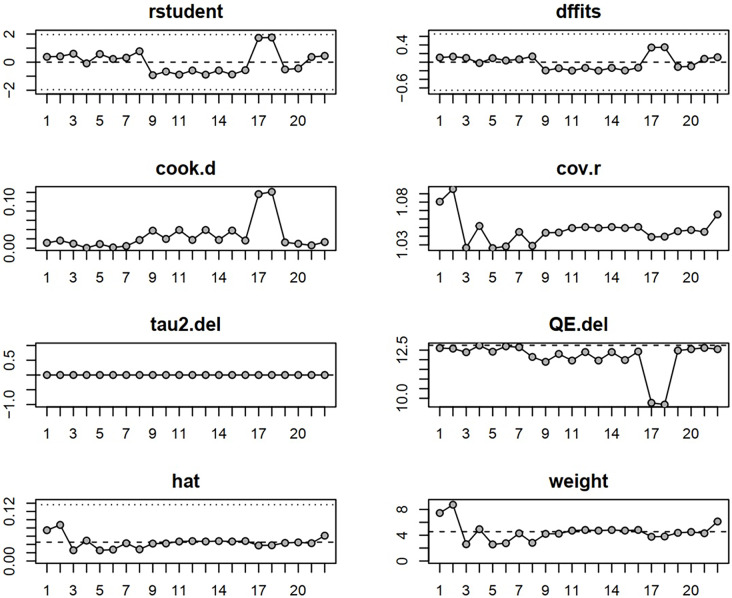
Influence analysis for T.

**Figure 6:**
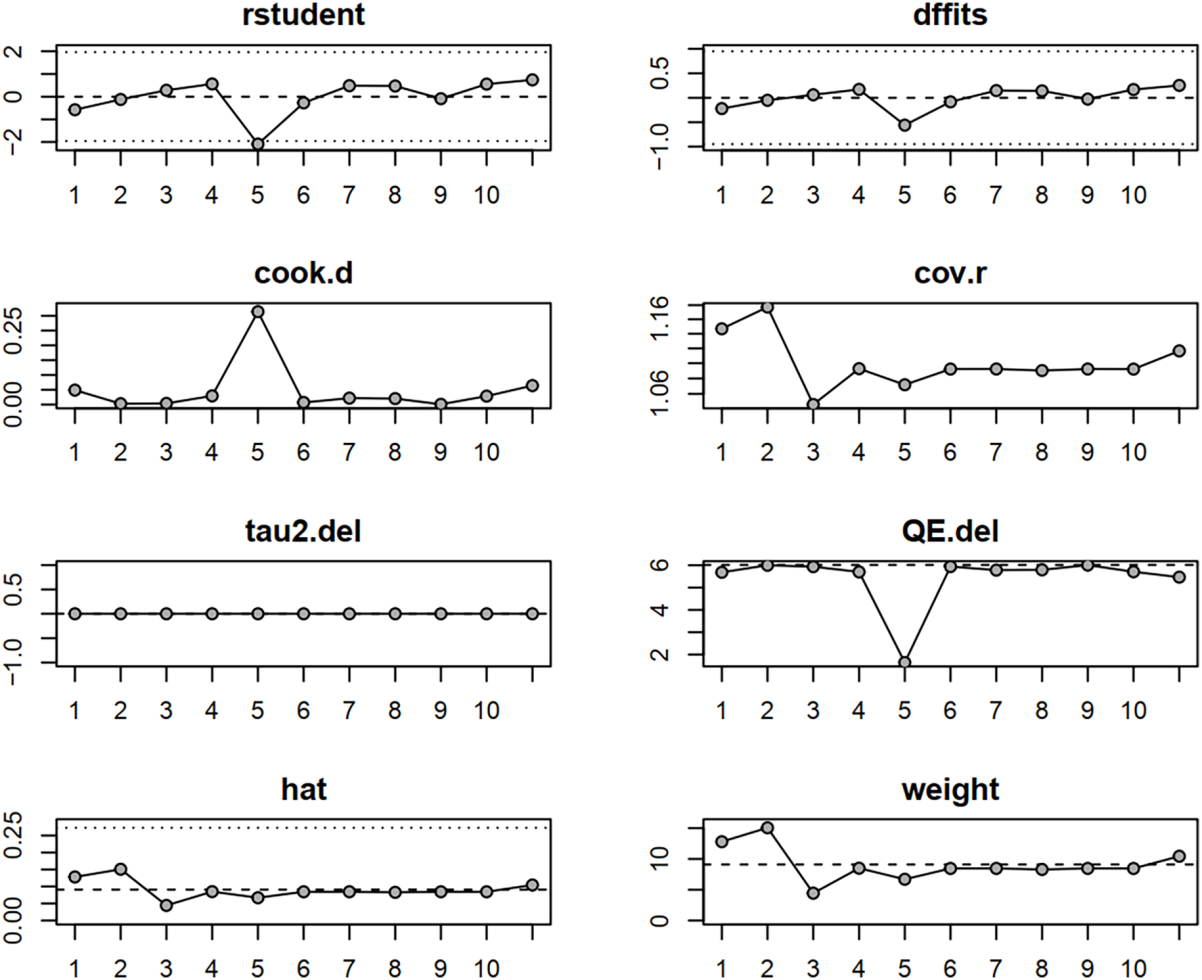
Influence analysis for FSH.

**Figure 7:**
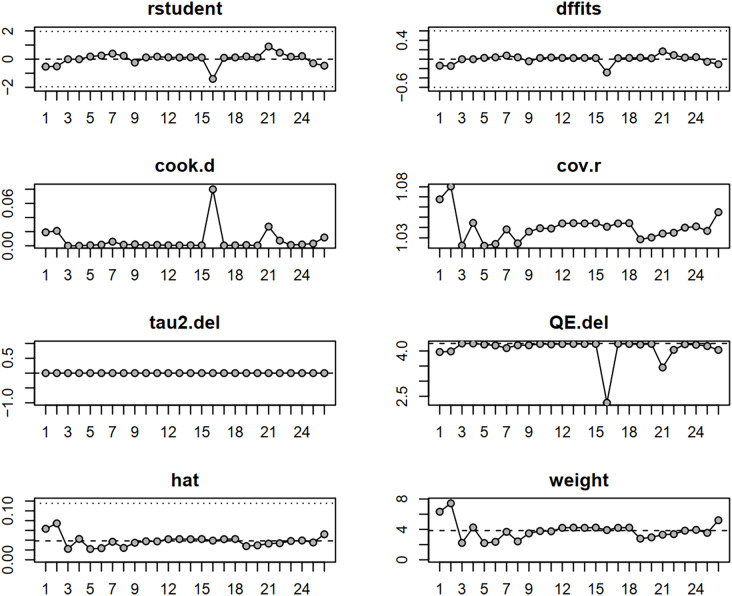
Influence analysis for BMI.

Influential analysis was conducted to assess whether any potential outliers disproportionately affected the pooled estimates. The diagnostic results indicated that no influential studies or outliers were detected for T (Fig. 5), FSH (Fig. 6), or BMI (Fig. 7), suggesting that the findings for these parameters were robust.

### Sensitivity analysis

To evaluate the stability of our findings, a leave-one-out sensitivity analysis was conducted. The results demonstrated that the pooled estimates for T remained consistently significant, with effect sizes ranging from −0.43 to −0.30 (Table 7). For FSH, the sensitivity analysis yielded pooled estimates ranging from 0.0064 to 0.1401, with all results consistently failing to reach statistical significance (Table 8). Regarding BMI, the pooled effect sizes fluctuated between −0.1719 and −0.1091 during the sensitivity analysis, with none of the iterations reaching statistical significance (Table 9). The fact that the omission of any individual study did not materially alter the direction or significance of the results confirms the robustness of our conclusions regarding the impact of exercise on these parameters.

**Table 7:** Sensitivity analysis results for T.

Removed study	K-remaining	Effect size	95% CI		*P*
			Lower	Upper	
Kiel et al. (2022)	20	−0.4320	−0.7050	−0.1590	0.0037
Vizza et al. (2016)	21	−0.3563	−0.6269	−0.0856	0.0125
Almenning et al. (2015)	20	−0.3547	−0.6334	−0.0761	0.0153
Sverrisdottir et al. (2009)	21	−0.3514	−0.6228	−0.0800	0.0138
Kumari et al. (2025)	21	−0.3508	−0.6318	−0.0699	0.0169
Giallauria et al. (2008)	21	−0.3504	−0.6380	−0.0628	0.0194
Vigorito et al. (2007)	21	−0.3486	−0.6335	−0.0637	0.0190
Nasiri et al. (2025)	21	−0.3470	−0.6241	−0.0700	0.0167
Turan et al. (2015)	21	−0.3455	−0.6229	−0.0682	0.0172
Jedel et al. (2011)	21	−0.3331	−0.6137	−0.0526	0.0223
Philbois et al. (2022)	20	−0.3081	−0.5963	−0.0198	0.0375
Ribeiro et al. (2021b)	20	−0.2961	−0.5792	−0.0130	0.0413
Lopes et al. (2018)	20	−0.2956	−0.5749	−0.0162	0.0392
Ribeiro et al. (2021a)	20	−0.2953	−0.5778	−0.0128	0.0413
Ribeiro et al. (2020)	20	−0.2953	−0.5778	−0.0128	0.0413

**Note:**

K, Effect size quantity.

**Table 8:** Sensitivity analysis results for FSH.

Removed study	K-remaining	Effect size	95% CI		*P*
			Lower	Upper	
Kumari et al. (2025)	10	0.0064	−0.3868	0.3995	0.9715
Jedel et al. (2011)	10	0.0199	−0.3691	0.4088	0.9104
Ribeiro et al. (2021b)	9	0.0223	−0.3937	0.4384	0.9045
Wu et al. (2021)	10	0.0247	−0.3638	0.4132	0.8888
Ribeiro et al. (2020)	9	0.0364	−0.3797	0.4524	0.8453
Sverrisdottir et al. (2009)	10	0.0380	−0.3426	0.4186	0.8265
Giallauria et al. (2008)	10	0.0565	−0.3472	0.4602	0.7588
Vigorito et al. (2007)	10	0.0843	−0.3141	0.4827	0.6437
Turan et al. (2015)	10	0.1401	−0.2450	0.5253	0.4318

**Note:**

K, Effect size quantity.

**Table 9:** Sensitivity analysis results for BMI.

Removed study	K-remaining	Effect size	95%CI		*P*
			Lower	Upper	
Kiel et al. (2022)	24	−0.1719	−0.4204	0.0765	0.1656
Philbois et al. (2022)	24	−0.1510	−0.4009	0.0989	0.2239
Turan et al. (2015)	25	−0.1507	−0.3947	0.0932	0.2145
Almenning et al. (2015)	24	−0.1498	−0.3954	0.0959	0.2198
Lopes et al. (2018)	24	−0.1490	−0.3985	0.1006	0.2294
Benham et al. (2021)	24	−0.1481	−0.3953	0.0990	0.2276
Ribeiro et al. (2021a)	24	−0.1480	−0.3988	0.1028	0.2345
Ribeiro et al. (2020)	24	−0.1480	−0.3988	0.1028	0.2345
Ribeiro et al. (2021b)	24	−0.1474	−0.3982	0.1034	0.2364
Vizza et al. (2016)	25	−0.1462	−0.3886	0.0962	0.2252
Sverrisdottir et al. (2009)	25	−0.1418	−0.3839	0.1004	0.2387
Jedel et al. (2011)	25	−0.1417	−0.3864	0.1030	0.2436
Costa et al. (2018)	25	−0.1365	−0.3802	0.1072	0.2590
Mohammadi, Monazzami & Alavimilani (2023)	25	−0.1354	−0.3791	0.1084	0.2631
Kumari et al. (2025)	25	−0.1293	−0.3752	0.1166	0.2887
Vigorito et al. (2007)	25	−0.1258	−0.3732	0.1215	0.3042
Giallauria et al. (2008)	25	−0.1250	−0.3739	0.1239	0.3102
Wu et al. (2021)	25	−0.1091	−0.3533	0.1352	0.3660

**Note:**

K, Effect size quantity.

### Risk of bias assessment results for the included studies

**Figure 8:**
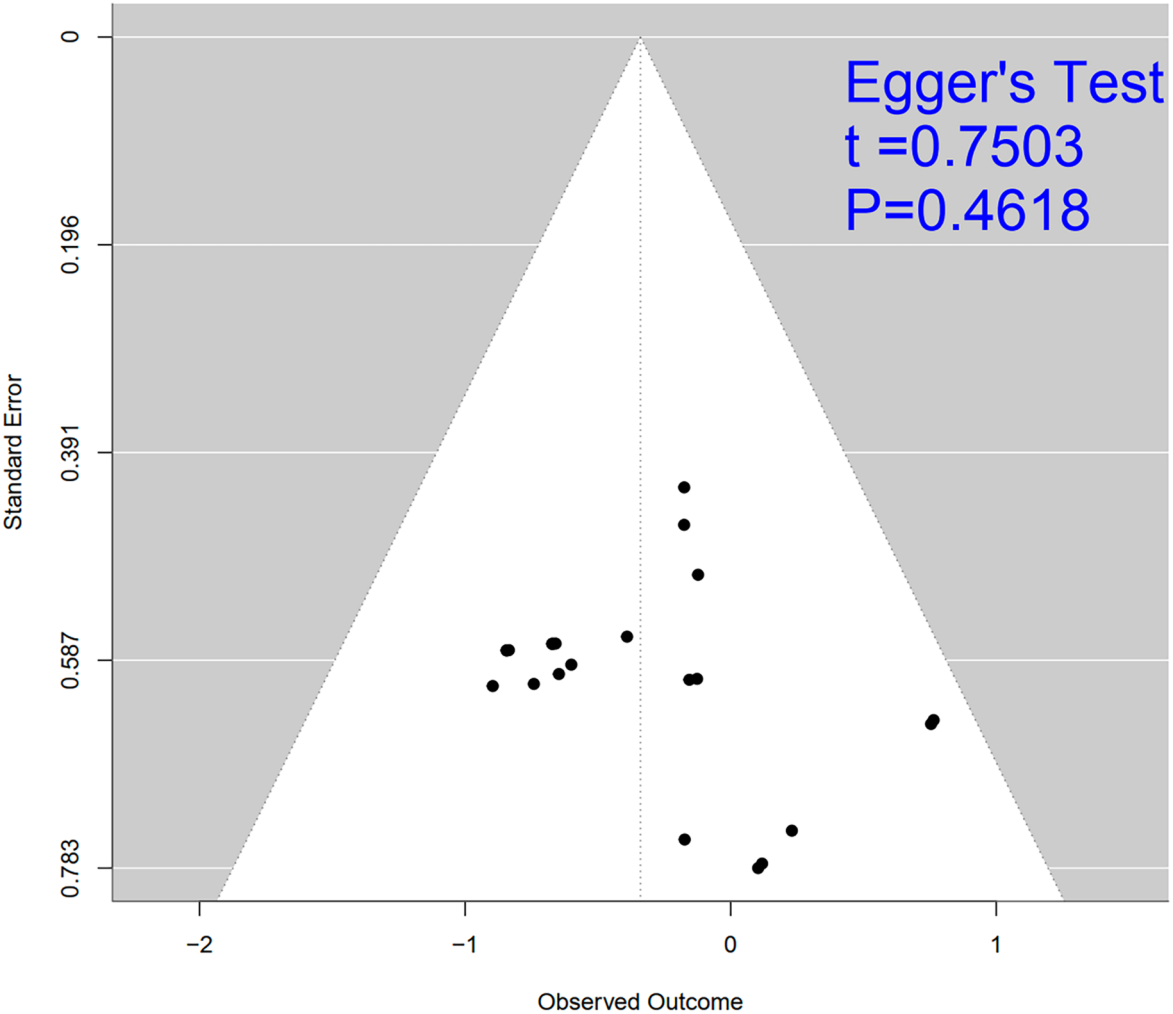
Funnel plot of publication bias for T.

**Figure 9:**
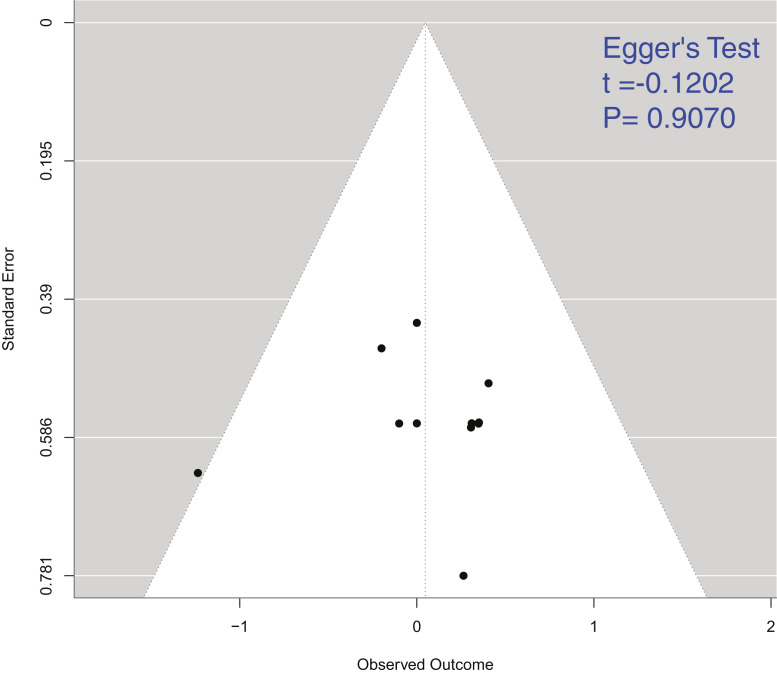
Funnel plot of publication bias for FSH.

**Figure 10:**
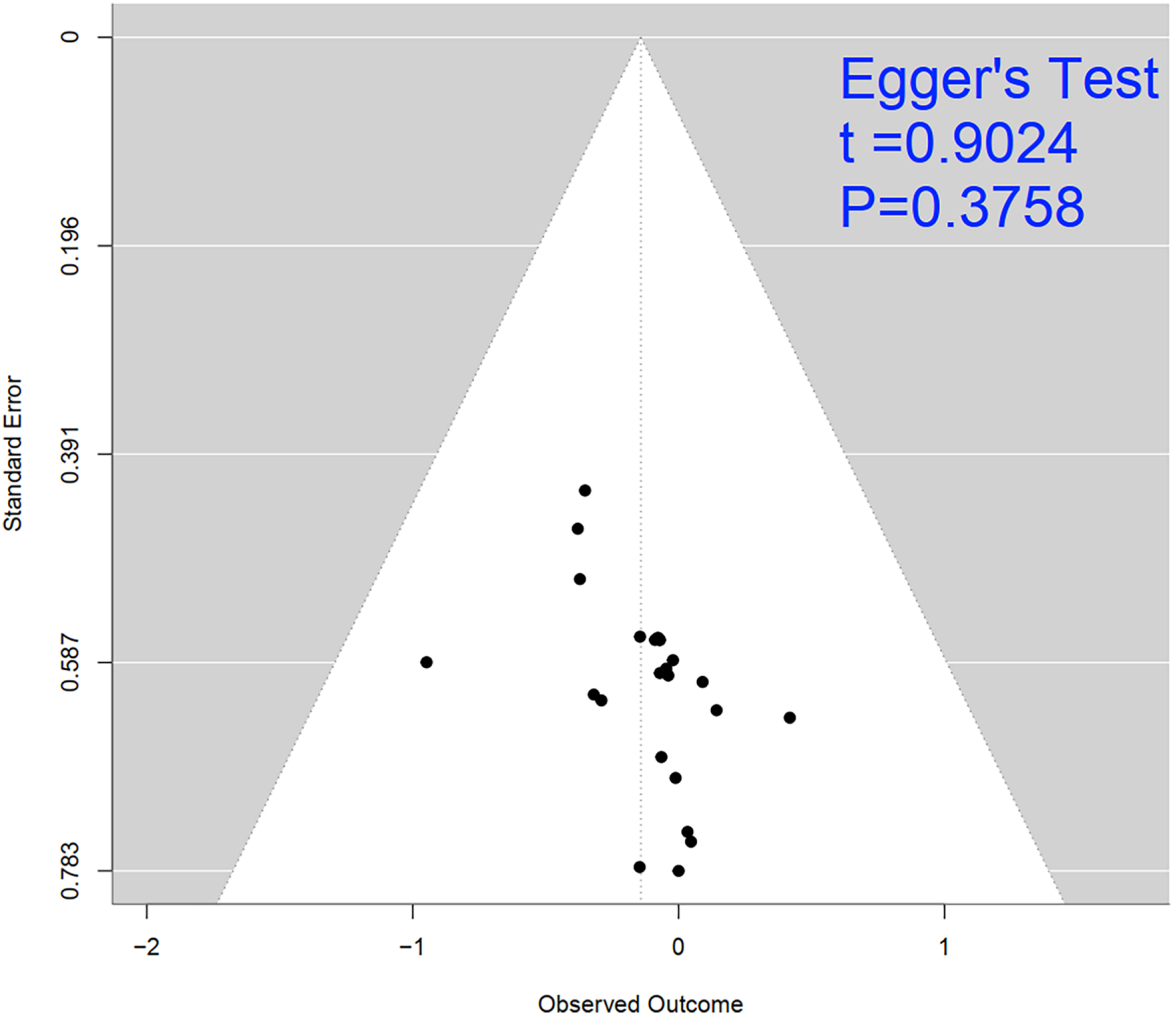
Funnel plot of publication bias for BMI.

The assessment of publication bias for studies reporting on T, FSH and BMI is illustrated in Figs. 8, 9, and 10, respectively. Visual inspection of the funnel plots (and/or statistical testing) revealed no evidence of significant publication bias across these three parameters.

### Quality evaluation

According to the GRADE criteria, the certainty of evidence for T, FSH, and BMI was not downgraded for publication bias, inconsistency, or indirectness. However, the evidence was downgraded by one level each for imprecision and risk of bias (study limitations). Consequently, the overall quality of evidence was categorized as low (Table 10).

**Table 10:** Evidence quality grading evaluation.

Outcome measures	Included studies	Factors influencing evidence quality evaluation					Quality assessment of evidence
		Publication bias in research	Measurement imprecision	Study limitations and constraints	Heterogeneity and inconsistency	Clinical indirectness	
T	15	Insignificant	Severe	Severe	Insignificant	Insignificant	Low quality
FSH	5	Insignificant	Severe	Severe	Insignificant	Insignificant	Low quality
BMI	18	Insignificant	Severe	Severe	Insignificant	Insignificant	Low quality

**Note:**

T, Testosterone; FSH, follicle-stimulating hormone; BMI, body mass index.

## Discussion

This three-level meta-analysis, incorporating 19 eligible studies, was conducted to evaluate the impact of exercise interventions on the reproductive endocrine profiles of adult patients with polycystic ovary syndrome (PCOS). The findings indicated that exercise exerted a favorable effect on T levels, whereas no significant improvements were observed for FSH or BMI.

In view of the mean PEDro score of 5.3 (moderate quality) and the low certainty of evidence—downgraded for imprecision and study limitations—potential biases arising from limited blinding and allocation concealment warrant careful consideration. Given that exercise interventions necessitate active participant engagement and diverse control modalities, blinding participants and therapists remains inherently challenging. Furthermore, the exclusive inclusion of published literature may introduce publication bias, while the substantial heterogeneity in exercise protocols, compounded by suboptimal allocation concealment, could exacerbate baseline imbalances or selection bias. These factors, alongside self-reporting errors and the subjectivity of hormonal assays, may systematically inflate the perceived efficacy of exercise on PCOS, necessitating a cautious interpretation of the findings. Additionally, the restricted number of effect sizes precluded a comprehensive analysis of all outcomes or specific PCOS phenotypes. Consequently, current evidence remains insufficient to establish practical guidelines for exercise intensity or duration, highlighting the urgent need for further research to refine evidence-based exercise prescriptions.

Our findings demonstrate that exercise intervention exerts a favorable impact on T levels, a phenomenon potentially mediated by the modulation of inflammatory pathways. Patients with PCOS frequently exhibit elevated circulating plasma levels of tumor necrosis factor (TNF)-α (Jakubowska et al., 2008). Exercise has been shown to suppress TNF-α production, thereby ameliorating insulin resistance and hyperinsulinemia; this cascade subsequently attenuates ovarian androgen synthesis, facilitating a detectable reduction in T levels (Barber & Franks, 2021). Although certain literature reports exercise to be ineffective in modulating T (Kelly dos Santos et al., 2020), such discrepancies may stem from limited sample sizes. While a previous meta-analysis on aerobic exercise synthesized data from only five studies (*n* = 342), the present study incorporated 10 studies encompassing 698 participants. Crucially, the application of a three-level meta-analytic framework in our research allowed for a more precise handling of dependencies between effect sizes, thereby bolstering statistical power and the overall robustness of the results (Pustejovsky & Tipton, 2022). Subgroup analysis in this study also found that aerobic exercise may be more beneficial for improving testosterone (T) levels in PCOS patients, and this trend is more obvious in patients with a BMI of 25–29.9 and a mean age of 26–30. The possible mechanism is that aerobic exercise improves fat oxidation capacity in PCOS patients through changes in muscle fiber types and mitochondrial density (van der Zwaard et al., 2016). While reducing weight, it regulates the neuroendocrine system, affects the function of the hypothalamus and pituitary, and then regulates ovarian androgen secretion (Murphy & Febbraio, 2026). A BMI of 25–29.9 is classified as overweight but not yet obese. From this perspective, it is speculated that when patients with a BMI ≥ 30 want to achieve a clinically significant BMI reduction (≥5%), they may need longer intervention periods under the same weekly exercise duration and intensity (Swift et al., 2018). For patients aged 26–30, due to the longer disease course and more persistent insulin resistance, exercise may reduce hyperinsulinemia by improving insulin signaling pathways, thereby decreasing androgen secretion (Huang-Doran et al., 2021). However, the differences between these subgroups lack statistical support, and these explanations remain exploratory and require further validation.

Our findings indicate that exercise intervention yields no significant impact on follicle-stimulating hormone (FSH) levels or body mass index (BMI) in patients with PCOS, with the latter finding diverging from previous reports (Kelly dos Santos et al., 2020). This discrepancy regarding FSH may stem from the pathophysiological hallmark of PCOS—follicular arrest (polycystic morphology)—wherein hyperandrogenism and insulin resistance impede normal follicular maturation. Consequently, FSH levels often remain within a low-to-normal range despite functional impairment (Zeng et al., 2020). Although exercise confers metabolic and endocrine benefits, FSH dynamics are primarily governed by the rhythmic pulsatility of the hypothalamic-pituitary-ovarian (HPO) axis, follicular status, and individual phenotypes. Short-term interventions may thus be insufficient to induce stable, statistically significant alterations in FSH secretion (Dewailly et al., 2016). Regarding BMI, obesity is known to exacerbate the metabolic and reproductive sequelae of PCOS by augmenting inflammatory adipokines and compensatory hyperinsulinemia, which collectively promote lipogenesis while inhibiting lipolysis (Glueck & Goldenberg, 2019). Importantly, the therapeutic efficacy of exercise may not be immediately reflected by BMI reduction. As BMI fails to differentiate between adiposity reduction and lean mass accretion and is subject to age-related and ethnic variability (Shanks, Bruening & Yaroch, 2025), it remains an insensitive marker of body composition changes. Consistent with recommendations from the American Medical Association (AMA), our findings underscore that integrating measures of adiposity and skeletal muscle mass with BMI is essential for a more accurate assessment and management of obesity in this population (Ross et al., 2020; Shanks, Bruening & Yaroch, 2025).

## Conclusions

In conclusion, exercise interventions exert a favorable impact on serum testosterone levels in adult patients with polycystic ovary syndrome.

## Supplemental Information

10.7717/peerj.21507/supp-1 Supplemental Information 1 Raw Data.

10.7717/peerj.21507/supp-2 Supplemental Information 2 PRISMA checklist.

## Abbreviation list

PCOS: Polycystic Ovary Syndrome
ASIR: Age-Standardized Incidence Rate
ASPR: Age-Standardized Prevalence Rate
T: Testosterone
FSH: Follicle-Stimulating Hormone
LH: Luteinizing Hormone
E_2_: Estradiol
AMH: Anti-Müllerian Hormone
PRL: Prolactin
P: Progesterone
BMI: Body Mass Index
RCT: Randomized Controlled Trial
PEDro: Physiotherapy Evidence Database
SMD: Standardized Mean Difference
EPOC: Excess Post-exercise Oxygen Consumption
GnRH: Gonadotropin-Releasing Hormone

## Additional Information and Declarations

### Data Availability

The following information was supplied regarding data availability:

This is a Systematic review/Meta-analysis.
