# Supplementary material for: Effects of exercise on reproductive endocrine hormones in adult patients with polycystic ovary syndrome: a systematic review and three-level meta-analysis
Source: PeerJ. 2026 Jul 29;14:e21507. doi: 10.7717/peerj.21507 (PMC13428543; doi:10.7717/peerj.21507)
Supplement: Supplemental Information 1 [file peerj-14-21507-s001.docx]

**Table 13.Meta-Analysis Data Extraction Sheet**

| Country where the study was conducted,  First author, Year of publication | Outcome Measure (Unit) | Intervention group | | | Control group | | |
| --- | --- | --- | --- | --- | --- | --- | --- |
|  |  | Mean Difference | Difference SD | n | Mean Difference | Difference SD | n |
| Italy, Carlo Vigorito, 2007 | BMI（kg/m^2^） | -1.3000 | 2.9000 | 45 | -0.1000 | 3.3601 | 45 |
|  | FSH（IU/L） | -0.2000 | 1.5395 | 45 | 0.1000 | 1.4422 | 45 |
|  | LH（IU/L） | -0.7000 | 3.2512 | 45 | -0.4000 | 3.0050 | 45 |
|  | PRL（ng/mL） | -0.2000 | 1.2124 | 45 | -0.1000 | 1.1533 | 45 |
|  | E₂（pmol/L） | -5.0000 | 31.2770 | 45 | -2.0000 | 27.6642 | 45 |
|  | P（nmol/L） | -0.1000 | 0.3606 | 45 | -0.1000 | 0.5568 | 45 |
|  | T（nmol/L） | -0.2000 | 0.6557 | 45 | -0.1000 | 0.4583 | 45 |
| Italy, Francesco Giallauria, 2008 | BMI（kg/m^2^） | -1.3000 | 2.7221 | 62 | -0.2000 | 3.4511 | 62 |
|  | FSH（IU/L） | -0.5000 | 5.4507 | 62 | -0.5000 | 5.3000 | 62 |
|  | LH（IU/L） | -0.6000 | 8.0542 | 62 | -0.2000 | 7.9731 | 62 |
|  | PRL（mU/L） | -6.0000 | 20.3517 | 62 | -4.0000 | 20.6502 | 62 |
|  | E₂（pmol/L） | -4.2000 | 31.6474 | 62 | -2.2000 | 33.1311 | 62 |
|  | P（nmol/L） | -0.1200 | 0.3606 | 62 | -0.0500 | 0.2646 | 62 |
|  | T（nmol/L） | -0.3000 | 1.2124 | 62 | -0.1000 | 1.0536 | 62 |
| Sweden, Elisabet Stener-Victorin, 2009 | BMI（kg/m^2^） | -0.4000 | 4.8000 | 5 | 0.5000 | 6.2000 | 6 |
|  | LH（IU/L） | -1.0000 | 2.8618 | 5 | -4.5000 | 10.0817 | 6 |
|  | FSH（IU/L） | -0.5000 | 1.6093 | 5 | -0.9000 | 1.1790 | 6 |
|  | T（nmol/L） | 0.0000 | 0.7937 | 5 | -0.1000 | 0.7550 | 6 |
| Sweden, Elizabeth Jedel, 2011 | BMI（kg/m^2^） | 0.0100 | 0.7000 | 30 | 0.1100 | 0.6300 | 15 |
|  | T（ng/mL） | -0.0400 | 0.1400 | 30 | 0.0100 | 0.0900 | 15 |
|  | E₂（pg/mL） | -18.8000 | 47.8000 | 30 | 11.2000 | 51.2000 | 15 |
|  | LH（IU/L） | -0.4500 | 4.3300 | 30 | -1.6300 | 7.9800 | 15 |
|  | FSH（IU/L） | 0.2600 | 1.5700 | 30 | -0.3100 | 1.6500 | 15 |
| Norway, Ida Almenning, 2015① | BMI（kg/m^2^） | 0.1000 | 4.8000 | 8 | 0.1000 | 5.2507 | 4 |
|  | T（nmol/L） | 0.0000 | 1.0149 | 8 | -0.1000 | 0.5000 | 4 |
|  | AMH（pmol/ L） | -11.4000 | 48.6096 | 8 | -5.4000 | 34.8062 | 4 |
| Norway, Ida Almenning, 2015② | BMI（kg/m^2^） | 0.4000 | 6.3647 | 8 | 0.1000 | 5.2507 | 5 |
|  | T（nmol/L） | -0.2000 | 0.5568 | 8 | -0.1000 | 0.5000 | 5 |
|  | AMH（pmol/ L） | -14.8000 | 26.4433 | 8 | -5.4000 | 34.8062 | 5 |
| Turkey,Volkan Turan,2015 | BMI（kg/m^2^） | -0.1000 | 1.0536 | 14 | -0.2000 | 1.1000 | 16 |
|  | FSH（IU/L） | -0.4000 | 0.2718 | 14 | -0.1000 | 0.2000 | 16 |
|  | LH（IU/L） | 1.7000 | 1.5362 | 14 | -0.1000 | 1.2530 | 16 |
|  | E₂（pmol/L） | 27.5000 | 12.2049 | 14 | 0.1000 | 3.8000 | 16 |
|  | T（nmol/L） | -0.1000 | 0.9000 | 14 | 0.0000 | 0.1732 | 16 |
| Australia, Lisa Vizza, 2016 | BMI（kg/m^2^） | 0.4000 | 12.3049 | 7 | 0.0000 | 9.4000 | 6 |
|  | T（nmol/L） | 0.2000 | 0.4359 | 7 | 0.1000 | 0.3606 | 6 |
| Brazil, EDUARDO CALDAS, 2018 | BMI（kg/m^2^） | -0.7000 | 4.3578 | 14 | 0.7000 | 5.0030 | 13 |
| Brazil, Iris Palma Lopes, 2018① | T（ng/dL） | -25.8000 | 47.9290 | 23 | 17.4000 | 45.4168 | 12 |
|  | BMI（kg/m^2^） | -0.2000 | 5.6507 | 23 | 0.2000 | 5.4028 | 12 |
| Brazil, Iris Palma Lopes, 2018② | T（ng/dL） | -20.3000 | 51.7490 | 22 | 17.4000 | 45.4168 | 12 |
|  | BMI（kg/m^2^） | 0.0000 | 4.8508 | 22 | 0.2000 | 5.4028 | 12 |
| Brazil, Victor Barbosa Ribeiro, 2019① | BMI（kg/m^2^） | -0.2000 | 5.6507 | 28 | 0.2000 | 5.3028 | 15 |
|  | T（ng/dL） | -24.0000 | 45.2106 | 28 | 14.0000 | 42.2256 | 15 |
|  | E₂（pg/mL） | 11.0000 | 60.3573 | 28 | 0.0000 | 23.0651 | 15 |
|  | LH（uUI/mL） | 0.5000 | 7.4646 | 28 | -2.4000 | 7.2993 | 15 |
|  | FSH（uIU/mL） | -0.6000 | 1.9672 | 28 | -0.4000 | 1.9079 | 15 |
| Brazil, Victor Barbosa Ribeiro, 2019② | BMI（kg/m^2^） | -0.2000 | 4.8000 | 29 | 0.2000 | 5.3028 | 15 |
|  | T（ng/dL） | -20.0000 | 53.0283 | 29 | 14.0000 | 42.2256 | 15 |
|  | E₂（pg/mL） | -2.0000 | 52.2590 | 29 | 0.0000 | 23.0651 | 15 |
|  | LH（uUI/mL） | -0.2000 | 4.8031 | 29 | -2.4000 | 7.2993 | 15 |
|  | FSH（uIU/mL） | 0.4000 | 2.8054 | 29 | -0.4000 | 1.9079 | 15 |
| Brazil, Victor B. Ribeiro, 2020① | BMI（kg/m^2^） | -0.2000 | 5.6507 | 28 | 0.2000 | 5.3028 | 15 |
|  | T（mg/dL） | -24.0000 | 45.2106 | 28 | 14.0000 | 42.2256 | 15 |
| Brazil, Victor B. Ribeiro, 2020② | BMI（kg/m^2^） | -0.2000 | 4.8000 | 29 | 0.2000 | 5.3028 | 15 |
|  | T（mg/dL） | -20.0000 | 53.0283 | 29 | 14.0000 | 42.2256 | 15 |
| China, Xia Wu, 2020 | BMI（kg/m^2^） | -2.5000 | 2.6665 | 19 | 0.5000 | 3.4771 | 19 |
|  | FSH（mUI/mL） | 0.2000 | 1.1358 | 19 | -0.2000 | 1.4107 | 19 |
|  | AMH（ng/mL） | -2.8000 | 4.5398 | 19 | -0.5000 | 5.3357 | 19 |
| Brazil, Victor Barbosa Ribeiro, 2021① | BMI（kg/m^2^） | -0.2600 | 5.6452 | 28 | 0.2400 | 5.3423 | 15 |
|  | T（ng/dL） | -24.0000 | 44.8106 | 28 | 13.4700 | 42.4872 | 15 |
|  | E₂（pg/mL） | 11.1700 | 60.3349 | 28 | 0.1900 | 22.9451 | 15 |
|  | LH（uUI/mL） | 0.5300 | 7.4287 | 28 | -2.4700 | 7.3001 | 15 |
|  | FSH（uIU/mL） | -0.5400 | 2.0031 | 28 | -0.4700 | 1.8938 | 15 |
| Brazil, Victor Barbosa Ribeiro, 2021② | BMI（kg/m^2^） | -0.1400 | 4.7903 | 29 | 0.2400 | 5.3423 | 15 |
|  | T（ng/dL） | -19.8900 | 52.9155 | 29 | 13.4700 | 42.4872 | 15 |
|  | E₂（pg/ml） | -2.0300 | 52.3963 | 29 | 0.1900 | 22.9451 | 15 |
|  | LH（uUI/ml） | -0.1400 | 4.8063 | 29 | -2.4700 | 7.3001 | 15 |
|  | FSH（uIU/ml） | 0.3800 | 2.8376 | 29 | -0.4700 | 1.8938 | 15 |
| Canada, Jamie L.Bham, 2021① | BMI（kg/m^2^） | 0.1000 | 8.3420 | 11 | 0.2000 | 8.6637 | 7 |
| Canada, Jamie L.Bham, 2021② | BMI（kg/m^2^） | -0.4000 | 9.0067 | 12 | 0.2000 | 8.6637 | 8 |
| Norway, IDA A.KIEL, 2022① | BMI（kg/m^2^） | 0.9000 | 4.9554 | 16 | -1.4000 | 5.8835 | 10 |
|  | T（nmol/L） | 0.3000 | 0.6619 | 16 | -0.2000 | 0.6062 | 10 |
|  | AMH（pmol/L） | -1.3000 | 24.4506 | 16 | -4.7000 | 32.3700 | 10 |
| Norway, IDA A.KIEL, 2022② | BMI（kg/m^2^） | -0.5000 | 6.2385 | 17 | -1.4000 | 5.8835 | 10 |
|  | T（nmol/L） | 0.3000 | 0.6496 | 17 | -0.2000 | 0.6062 | 10 |
|  | AMH（pmol/L） | 14.0000 | 24.0394 | 17 | -4.7000 | 32.3700 | 10 |
| Brazil, Stella V.Philbois, 2022① | BMI（kg/m^2^） | -0.2000 | 6.7816 | 25 | 0.1000 | 5.4000 | 12 |
|  | T（ng/dL） | -16.9000 | 35.2382 | 25 | 7.4000 | 39.8752 | 12 |
| Brazil, Stella V.Philbois, 2022② | BMI（kg/m^2^） | 0.0000 | 4.2000 | 25 | 0.1000 | 5.4000 | 13 |
|  | T（ng/dL） | -20.2000 | 47.3975 | 25 | 7.4000 | 39.8752 | 13 |
| Iran, Somayeh Mohammadi, 2023 | BMI（kg/m^2^） | -1.1300 | 4.4851 | 14 | 0.0800 | 2.6514 | 14 |
| Iran, Masoud Nasiri, 2025 | AMH（ng/mL） | -0.0500 | 1.2750 | 15 | 0.0500 | 1.3253 | 15 |
|  | T（ng/mL） | -0.0600 | 0.2307 | 15 | 0.0200 | 0.2400 | 15 |
| India, Deepika Kumari, 2025 | BMI（kg/m^2^） | -0.6850 | 5.3061 | 32 | 1.1590 | 4.4203 | 29 |
|  | FSH（mIU/mL） | 0.7750 | 1.2513 | 32 | 0.2600 | 1.2601 | 29 |
|  | LH（mIU/mL） | -1.2510 | 1.6851 | 32 | 0.0150 | 1.8713 | 29 |
|  | AMH（ng/mL） | -1.7200 | 2.8813 | 32 | -0.2470 | 2.3906 | 29 |
|  | T（ng/dL） | -1.4350 | 6.2079 | 32 | -0.6820 | 5.9252 | 29 |

T=Testosterone; FSH=Follicle-Stimulating Hormone; LH=Luteinizing Hormone; E₂=Estradiol; AMH=Anti-Müllerian Hormone; PRL=Prolactin; P=Progesterone; BMI=Body Mass Index.
